# Updates in Alzheimer's disease: from basic research to diagnosis and therapies

**DOI:** 10.1186/s40035-024-00432-x

**Published:** 2024-09-04

**Authors:** Enjie Liu, Yao Zhang, Jian-Zhi Wang

**Affiliations:** 1 Department of Pathology, The First Affiliated Hospital of Zhengzhou University, Zhengzhou, 450052 China; 2 Department of Endocrine, Liyuan Hospital, Key Laboratory of Ministry of Education for Neurological Disorders, Tongji Medical College, Huazhong University of Science and Technology, Wuhan, 430077 China; 3 Department of Pathophysiology, Key Laboratory of Ministry of Education for Neurological Disorders, School of Basic Medicine, Tongji Medical College, Huazhong University of Science and Technology, Wuhan, 430030 China; 4 Co-Innovation Center of Neuroregeneration, Nantong University, Nantong, 226000 China

**Keywords:** Alzheimer’s disease, Neurodegeneration, Tau, β-Amyloid, Diagnosis, Drug development

## Abstract

Alzheimer’s disease (AD) is the most common neurodegenerative disorder, characterized pathologically by extracellular deposition of β-amyloid (Aβ) into senile plaques and intracellular accumulation of hyperphosphorylated tau (pTau) as neurofibrillary tangles. Clinically, AD patients show memory deterioration with varying cognitive dysfunctions. The exact molecular mechanisms underlying AD are still not fully understood, and there are no efficient drugs to stop or reverse the disease progression. In this review, we first provide an update on how the risk factors, including *APOE* variants, infections and inflammation, contribute to AD; how Aβ and tau become abnormally accumulated and how this accumulation plays a role in AD neurodegeneration. Then we summarize the commonly used experimental models, diagnostic and prediction strategies, and advances in periphery biomarkers from high-risk populations for AD. Finally, we introduce current status of development of disease-modifying drugs, including the newly officially approved Aβ vaccines, as well as novel and promising strategies to target the abnormal pTau. Together, this paper was aimed to update AD research progress from fundamental mechanisms to the clinical diagnosis and therapies.

## Introduction

Alzheimer's disease (AD) is the most common neurodegenerative disorder mainly affecting individuals aged over 65 [1]. Pathologically, AD is characterized by extracellular deposition of β-amyloid (Aβ) and intracellular accumulation of hyperphosphorylated tau (pTau), forming senile plaques and neurofibrillary tangles (NFTs), respectively [2–4]. Clinically, AD is associated with memory deterioration, often accompanied by aphasia, agnosia, impairment of visuospatial abilities, difficulties in abstract thinking and problem-solving, as well as personality and behavioral changes [5].

Over 95% of the AD cases have sporadic onset, in which the etiology and pathogenesis are still not clearly clarified. The apolipoprotein E (*APOE*) gene is considered the most significant genetic risk factor of AD [6–8]. Individuals carrying one or two *APOE *ε4 (*APOE4*) alleles have ~ 3.2 and 8–12 times higher risk of AD. Additionally, factors such as low education, smoking, estrogen reduction, high blood pressure, type-2 diabetis millitus, high cholesterol, and increased homocysteine levels are also associated with an increased risk of AD [9].

Familial AD (FAD) accounts for less than 5% of the cases and has onset before the age 65. With an autosomal-dominant inheritance pattern, FAD is primarily caused by mutations in genes for the amyloid precursor protein (*APP*) located on chromosome 21, *PSEN1* on chromosome 14, and *PSEN2* on chromosome 1. Almost all individuals carrying mutations in *APP* and *PSEN1* are destined to develop AD, and those carrying mutations in *PSEN2* have ~ 95% AD probability [10, 11].

Regarding the molecular mechanisms underlying AD pathologies and behavioral changes, the amyloid cascade hypothesis still dominates the field [12]. It is recognized that Aβ pathology may occur long before the formation of amyloid plaques; and soluble Aβ, particularly Aβ oligomers, plays a crucial role in disease progression [13]. The roles of neuroinflammation and abnormal activation of glial cells in mediating Aβ toxicity have received great attention [14]. Additionally, impaired Aβ clearance is an important mechanism leading to Aβ accumulation in the brain [15, 16].

In recent years, the critical role of tau in AD pathogenesis has been confirmed. Studies have been carried out to investigate the non-microtubule assembly and stabilizing functions of tau (such as regulating cell viability, acting as an acetyltransferase, etc.), tau release from neurons and propagation in different brain regions, the interplay of different post-translational modifications on tau proteins, and cleaved tau as "seeds" in tau aggregation, etc. [17]. In addition, *APOE* gene polymorphisms and chronic neuroinflammation induced by pathogen infections and glial activation in AD have also attracted attention.

The clinical diagnosis of AD has been mainly relying on doctors’ subjective evaluation based on the application of multiple psychometric scales, and biomarkers from the brain or cerebrospinal fluid (CSF) can help confirm the diagnosis [18]. In recent years, increasing studies have been aimed at searching for peripheral biomarkers from AD patients or populations with high AD risk, such as the aged populations and patients with type-2 diabetes mellitus (T2DM) [19–25]. The commonly supplied AD therapeutic drugs include cholinesterase inhibitors and *N*-methyl-*D*-aspartate (NMDA) receptor antagonists, which can only temporally improve the symptoms but not cure the disease. Most recently, several Aβ-targeting drugs have gained official approval [26–28], and development of therapeutics targeting tau or pTau is also emerging [29–31]. Currently, animal models that can faithfully replicate the pathologies and behavioral changes observed in AD patients are still lacking, which may be one of the major obstacles to more efficient drug development.

Over all, research on AD in the recent four decades has greatly enhanced our understanding of AD pathogenesis and provided new potential tools for AD diagnosis and treatment, though many questions remain to be addressed. This review will focus on the aspects that have received widespread attention and made significant progress, including major risk factors, roles of Aβ and tau in AD pathogenesis, commonly used experimental models, and advances in the diagnosis of and disease-modifying drug development for AD. Current challenges or speculations/suggestions in the related topics are also discussed.

## Major risk factors of AD

There are over 20 risk factors, including age, genetic mutation or variants, traumatic brain injury, and co-morbidities such as diabetes and infection. In light of recent progress, below we mainly review APOE and brain infections as risk factors for AD.

### Role of APOE in AD

#### Biology of APOE isoforms and their receptors

APOE consists of 299 amino acids with a molecular weight of ~ 34 kDa. APOE has three isoforms (APOE2, APOE3, and APOE4) encoded by three alleles (ε2, ε3, and ε4). The three isoforms differ in two amino acids at positions 112 and 158 (APOE2: Cys112 and Cys158, APOE3: Cys112 and Arg158, and APOE4 Arg112 and Arg158) [32, 33]. Although studies have shown that these single amino acid polymorphisms can substantially influence the structure and function of APOE by modulating its binding to lipids and receptors, it remains unclear how this small amino acid difference leads to such profound effects on AD [34]. The secondary structure of the APOE proteins includes α-helices, β-sheets, β-turns, and irregular structures with two distinct regions: the receptor-binding region in the N-terminal and the lipid-binding region in the C-terminal [35].

APOE is known to mediate lipid transport and utilization, thereby involved in neural structure, functions, injury and repair. When the neuronal axons are injured, the distal fibers undergo typical structural and functional changes. The residual fibers with myelin sheaths undergo degeneration, which become rich in cholesterol and phospholipids (Sudanophilic bodies) [36]. During the initial phase of neuroregeneration, a significant lipid accumulation occurs at the site of injury, and macrophages migrate to the injury site, where they synthesize and secrete APOE, capturing lipid bodies and storing them in macrophages [37, 38]. The lipids carried by macrophages are utilized for axonal and myelin regeneration [39]. Although highly specialized mature neurons lack the ability to divide and proliferate, intact axons can be induced to grow collateral branches and differentiate into synapses from the damaged neuronal fibers. For example, damage to the olfactory cortex results in the loss of approximately 60% of synaptic inputs to the granule cell layer of the hippocampus, but new synapses can be formed from the sprouting of surviving axons [40]. This compensatory process is completed in several months in parallel with increased APOE expression and enhanced APOE binding to low-density lipoprotein receptor (LDL-R) [41]. Homozygous *APOE* knockout mice exhibit age-dependent dendritic cytoskeletal breakdown and synaptic loss, emphasizing an indispensable role of APOE in the maintenance and reconstruction of the central nerves system.

There are at least three types of APOE receptor in the brain, including very low-density lipoprotein receptor (VLDL-R) [42], LDL-R [43], and low-density lipoprotein receptor-related protein (LRP) [44]. VLDL-R and LDL-R are predominantly located on the astrocyte membrane, while LRP is mainly distributed in neurons and activated astrocytes [45]. LRP accumulates in the sites of senile plaques, with a significant difference in the length of a tetranucleotide repeat sequence (TTTC)n upstream of the LRP gene between AD patients and healthy individuals. Both APOE and APP can bind to LRP. Any structural changes in LRP can affect the uptake and metabolism of APP, leading to Aβ overproduction [46]. Currently, the relationships of LDL-R and VLDL-R gene variants with AD are still controversial.

#### *APOE4* allele is a high-risk factor of AD

FAD is linked to the 19q13 chromosomal region, where the *APOE* gene is located. In the central nerves system, APOE is mainly expressed in astrocytes and contributes to a metabolic link between astrocytes and neurons. In the brains of AD patients, the level of APOE co-localized with senile plaques and NFTs in astrocytes is significantly increased compared to the control group [47]. The prevalence of AD has been strongly linked to *APOE* gene polymorphism. The *APOE4* allele is recognized as a high-risk factor for AD and the *APOE *ε3 (*APOE3*) allele is the most common allele and does not seem to influence the risk [48]. Reducing APOE4 in carriers is a therapeutic goal for AD [49]. Although there are conflicting reports [50], *APOE* ε2 (*APOE2*) is commonly considered as an AD protective and longevity allele [51]. *APOE2* gene therapy has been shown to reduce Aβ deposition and improve markers of neuroinflammation and neurodegeneration [52]. The proportion of *APOE2* in long-lived elderly European and American populations is almost twice that of the general life-span population, while the frequency of *APOE2* allele in AD patients is extremely low [53].

Within the central nervous system, APOE4 is produced by a variety of cell types under different conditions, posing a challenge for studying its roles in AD pathogenesis [54]. The evidence supporting *APOE4* as an AD risk factor is that *APOE4* increases the risk of both early- and late-onset AD [55–57]. Populations carrying one copy of *APOE4* allele have a 3–4-fold increased risk of late-onset AD, while the risk increases to 8–12 folds for those carrying two copies of *APOE4* [58–61]. Women with one *APOE4* allele display greater risk and earlier onset of AD compared with men [62, 63]. The follicle–stimulating hormone (FSH) in females with the *APOE4* but not the *APOE3* allele  increases the vulnerability to AD by activating the C/EBPβ/δ-secretase signaling [64]. In APOE4/C/EBPβ double transgenic mice, key AD pathologies appear in an age-dependent manner [65]. In contrast, APOE loss-of-function variants confer resistance to AD pathology [66].

Additionally, *APOE4* carriers have an earlier age of onset, who show AD symptoms at around 75 or 65 years of age, compared to the average onset age of 84 years [67, 68]. A meta-analysis revealed that increased frequency of *APOE4* allele is associated with increases of age-adjusted AD incidence, whereas no such relationship exists for *APOE2* and *APOE3* alleles [69]. The *APOE4* allele is also a susceptible factor for atherosclerosis [70], and AD patients often have vascular problems. Different from *APOE2* and *APOE3*, *APOE4* affects lipid transport and utilization, but its role in AD is not clear [71].

Microglia in *APOE4* knock-in mice exhibited significantly less brain surveillance (27%) compared to *APOE3* microglia at 6 months of age, and aging exacerbated this deficit [72]. APOE has the most enriched gene expression in neurodegenerative microglia. APOE4-mediated induction of ITGB8‒transforming growth factor-β (TGFβ) signaling impairs the neurodegenerative microglia response in AD via upregulation of microglial homeostatic checkpoints, including Inpp5d. Manipulating *APOE4* expression in the microglial cells significantly changes the quiescent state and the functions of the microglia [73–75]. Lipid accumulation induced by APOE4 impairs microglial surveillance of neuronal-network activity [76], and Aβ induces lipid droplet accumulation, tau phosphorylation and neurotoxicity in an APOE4-dependent manner [77]. APOE4 can impair neuron-astrocyte coupling of fatty acid metabolism, which could underlie the accelerated lipid dysregulation and energy deficits and increased AD risk for *APOE4* carriers [78]. APOE4 leads to neurovascular dysfunction and loss of integrity of the blood–brain barrier (BBB) [79].

#### Role of APOE4 on Aβ pathology

(1) APOE4 promotes Aβ aggregation

In human pluripotent stem cell-derived neurons expressing APOE4, both production and release of Aβ are significantly increased. APOE4 enhances the seeding properties of Aβ, promoting its aggregation and deposition [80, 81]. In APP transgenic mice, APOE4 can form a stable complex with Aβ that is resistant to degradation [82]. Removal of APOE4 results in the disappearance of Congo red-stained Aβ plaque-like structures, while reintroducing *APOE4* leads to the formation of senile plaques [83, 84]. AD patients carrying *APOE4* show a high level of Aβ oligomers in synapses, which leads to the recruitment and activation of microglia [85–87]. Thus, APOE4 may damage synapses by synergistically interacting with Aβ oligomers. The C-terminal 13-kDa fragment of APOE4 can bind to Aβ and thus promote the formation of highly toxic low-molecular-weight Aβ species [88].

(2) APOE4 inhibits Aβ degradation and clearance

The clearance of Aβ from the brain relies on APOE-mediated mechanisms. APOE2 or APOE3 can form complexes with Aβ and clear Aβ from brain via binding to VLDL-R and LRP1 on the BBB, predominantly via the VLDL-R pathway. Due to the lower internalization rate of APOE4-Aβ complexes mediated by VLDL-R compared to LRP1, the efficiency of Aβ clearance is lower in the presence of APOE4 than APOE2 and APOE3 [89]. A most recent study shows that IL-33 induces expression of vascular cell adhesion molecule-1 in microglia, which promotes microglial chemotaxis toward Aβ plaque-associated APOE, leading to Aβ clearance [90].

The phagocytic and degradative capacity of astrocytes and microglia towards Aβ is also influenced by the APOE genotype. Astrocytes expressing APOE4 show reduced uptake of Aβ42 compared to those expressing APOE3 [54]. In microglia, APOE promotes Aβ degradation via neprilysin and APOE4 exhibits the lowest ability among the three isoforms [54]. APOE-mediated cholesterol efflux facilitates Aβ transport to lysosomes and enhances the intracellular Aβ degradation by microglia, and this process is impaired in cells expressing APOE4 [91].

Compared to individuals without APOE4, AD patients with *APOE4* exhibit significantly reduced expression of Aβ-degrading enzymes, such as neprilysin and IDE, resulting in diminished Aβ degradation capacity [92]. In *APOE4* transgenic mice, brain injection of Aβ40 induces abundant Aβ deposition in the perivascular space of lymphatic-like vessels, suggesting involvement of perivascular lymphatic system [93].

#### APOE on tau-associated pathologies

Compared with Aβ, much less is known regarding the effect of APOE on tau. It was reported that the postmortem brains from individuals carrying two *APOE4* alleles have more tau aggregates than those carrying either one or no *APOE4* allele, and this effect is Aβ-dependent. In progressive supranuclear palsy (PSP), tau pathology is associated with *APOE4* [94]. The associations between synaptic density and tau pathology are regulated by the *APOE4* genotype [56].

(1) Effects of *APOE* gene polymorphism on tau

*APOE* gene knockout causes tau hyperphosphorylation and aggregation, with age-dependent deterioration of neuronal dendrites and microtubules, suggesting a crucial role of APOE in maintaining normal tau metabolism and microtubule stability [95, 96]. It is generally recognized that APOE3 may confer resilience to tauopathies [97], while APOE4, especially the C-terminal truncated form (APOE4 Δ272–299), promotes tau phosphorylation/aggregation and exacerbates neurodegeneration [98–100]. Expression of APOE4 in astrocytes disrupts tau uptake, trafficking and clearance [101]. With regard to APOE2, many studies have shown protective effects against amyloid-like pathology. However, there is still controversy on the effect of APOEε2 on tau [100, 102–104]. Some studies have reported that an increased level of APOE2 protein in the brain contributes to increased tau aggregation and behavioral impairment, and that APOE2 is positively correlated with the severity of tau pathology in patients with PSP [102, 104]. It is also reported that both *APOE3* and *APOE2* are protective and it is the absence of *APOE3* or *APOE2* rather than the presence of *APOE*4 that promotes tau pathologies.

A recent whole-exome sequencing study revealed an additional rare homozygous mutation of *APOE*3 (APOE3ch, R136S) as the potential protective factor against AD [105]. In vivo follow-up by PET imaging and postmortem studies revealed that the APOE3ch delayed AD onset for almost three decades beyond the expected age of onset [106]. APOE3ch expression alleviated Aβ deposition, tau pathology, astrogliosis, and cell death, with the mechanisms involving an increased myeloid cell phagocytosis [107, 108]. This resistance may be due to the reduced pathological interactions between APOE3Ch and heparan sulfate proteoglycans (HSPGs) [109].

(2) Effects of APOE receptors on tau

LRP1 can internalize tau and then mediate tau degradation in the lysosome. LRP1 has high affinity to tau at the microtubule-binding domain of tau, while phosphorylation of tau inhibits the associations between tau and LRP1 and thus decreases the internalization of extracellular tau proteins, which may play a role in the spreading of the pathological tau in the AD brains [110]. LRP1-mediated uptake of tau is also inhibited by APOE, and APOE4 is the most potent inhibitor, likely because of its higher affinity for LRP1 [111].

### Pathogenic microbial infection and AD

In recent years, the role of neuroinflammation in the occurrence and development of AD has received much attention. Various factors can induce chronic neuroinflammation through different mechanisms, promoting AD onset and progression [112].

#### Evidence supporting the role of microbial infection in AD

The hypothesis that AD may be caused by pathogenic infections was initially proposed by Dr. Oskar Fischer in 1907 [113]. In 1991, the DNA of herpes simplex virus type 1 (HSV-1, also known as human herpesvirus HHV-1) was detected in the brains of AD patients [114], and now HSV-1 has been experimentally confirmed to play a role in AD [115, 116]. Currently, increasing pathogens are being found to be associated with AD, such as herpesviruses HHV-1, HHV-2, HHV-3, HHV-5, HHV-6, and HHV-7, hepatitis C virus (HCV), chlamydia pneumoniae, spirochetes, periodontal bacteria, *Helicobacter pylori* (*H. pylori*), and intestinal microbiota. Antiviral drugs for herpesviruses can reduce the risk of dementia. These pathogens may enter the brain by directly crossing the BBB and/or the blood-cerebrospinal fluid barrier, or through the trigeminal system or the oral-nasal route. The pathogens may also cause inflammatory damage by secreting toxins that can enter the brain through the circulatory system [117].

####  Viral infection and AD

(1) Human herpesviruses

HSV-1 is a common neurotropic virus, with approximately 80% of the population carrying antibodies for HHV-1 [118, 119]. The levels of HHV-1 DNA in the brains of the elderly and AD patients are higher than that in the young people, which may be related to the age-related decline of immune function [120]. The titers of anti-HHV-1 antibodies in the CSF of the elderly and AD patients are also significantly increased [121]. APOE4 can modulate the severity of or susceptibility to microbial infections and promote HHV-1 neurotropic infection [122]. Neuronal cells infected with HHV-1 exhibit Aβ and tau aggregation [120, 123]. In 3D brain models of human induced neural stem cells that undergo differentiation and development, HSV-1 infection induces amyloid-like protein deposition, gliosis, neuroinflammation, and neural dysfunction [124].

HHV-2 infection leads to Aβ deposition, tau hyperphosphorylation, and inhibition of the non-amyloidogenic APP processing pathway [125, 126]. Epidemiological data show that exposure to *Toxoplasma gondii*, cytomegalovirus, or HSV-2 is associated with cognitive decline in the elder population [127]. Seropositivity for HHV-5 (cytomegalovirus, CMV) is associated with an increased risk of AD [128], and the levels of CMV antibodies are correlated with the severity of neurodegeneration. By measuring the peripheral blood leukocyte samples, the HHV-6 positive rate is 23% in AD patients and 4% in the controls; and 17% of AD patients are HHV-6-positive in brain samples. AD patients show elevated levels of HHV-6A and HHV-7 RNAs in multiple brain regions, which are correlated with plaque burden, tangle density, and the dementia severity.

(2) HCV

HCV infection is an independent risk factor for both AD and vascular dementia [129, 130]. Viruses may exert neurotoxic effects indirectly through systemic inflammation or directly by infecting the brain. HCV can cross the BBB and secrete high levels of cytokines such as IL-6 and TNF-α to induce toxic effects in the brain. In patients with hepatitis C, microglial activation positively correlates with cerebral metabolic changes [130].

#### Bacterial infection and AD

(1) *H. pylori*

*H. pylori* is a common resident bacterium in the stomach, infecting an estimated half of the global population [131]. In addition to its direct association with gastric ulcers and gastric cancer, *H. pylori* infection is closely linked to AD, atherosclerosis, hypertension, cerebral ischemia, and stroke [131]. AD patients show a significant increase in specific IgG levels against *H. pylori* in their blood and CSF [132]. Clinical studies have confirmed that the incidence of dementia is much higher in *H. pylori*-positive individuals than that in negative individuals. Experimental studies have also shown that the conditioned culture medium of *H. pylori* can promote tau hyperphosphorylation and Aβ overproduction.

(2) *Porphyromonas gingivalis*

*P. gingivalis* is the primary pathogen associated with chronic periodontitis. It can damage cells through its lipopolysaccharides (LPS), gingipains, and proteases produced by the bacterium. The bacteria and their molecules, such as outer membrane proteins, flagella proteins, fimbriae proteins, peptidoglycans, and proteases, can act as pathogen-associated molecular patterns. They interact with Toll-like receptors (TLRs) and induce the secretion of pro-inflammatory cytokines, leading to BBB disruption and neural damage. *P. gingivalis* has been detected in the brains of both AD and healthy individuals, suggesting that this bacterium may require synergistic interactions with other factors to promote AD [133].

Additionally, *Chlamydia pneumoniae* and spirochete bacteria have also been reported to be associated with AD. *C. pneumoniae* is a respiratory tract pathogen that can infect various types of brain cells and can exist within the inclusion bodies inside the cell, thereby escaping from immune recognition and lysosomal fusion. Specific DNA of *C. pneumoniae* has been detected in the AD brains by electron microscopy and immunohistochemistry [134]. However, due to the chronic course of AD, it is difficult to determine whether *C. pneumoniae* infection directly leads to AD or indirectly promotes AD progression through peripheral inflammation or respiratory dysfunction. Spirochete bacteria have also been detected in the CSF, blood, and brain tissues of some AD patients. Spirochetes are Gram-negative spiral bacteria with internal flagella that can invade the brain and establish latent and persistent infections [135]. They are the most neurotropic bacteria and can cause severe cerebrovascular pathology, cerebral hypoperfusion, brain dysfunction, and dementia.

#### Fungi and *T. gondii* in AD

Fungal DNA or proteins have been detected in the CSF and frozen brain tissues of AD patients, along with the presence of different antifungal antibodies. By using specific antibodies against fungi, researchers found that fungal infections exist in various brain areas, including cerebral cortex, cerebellum, olfactory cortex/hippocampus, and choroid plexus in AD patients. However, the role of fungal infections in AD is still uncertain [136].

Epidemiological studies have shown that olfactory dysfunction in patients with AD, multiple sclerosis, and schizophrenia is significantly associated with the elevated serum levels of anti-*T. gondii* IgG antibodies. Experimental research has revealed that chronic infection with *T. gondii* causes neuroinflammation [137].

#### Gut microbiota and AD

The human gut harbors bacteria, viruses, and fungi [138]. In healthy individuals, these microorganisms form a microbiota defensive barrier in the digestive tract. Recent studies reveal that alterations in gut microbiota are involved in various neurodegenerative diseases including AD [139].

Through 16S rRNA sequencing of fecal bacteria, significant differences in gut microbiota between APP/PS1 and wild-type mice have been observed [140]. The brain Aβ deposition in germ-free APP/PS1 mice is significantly lower than that in mice with normal gut microbiota. Transplantation of the AD feces significantly increased the brain Aβ level in the germ-free mice, while gut microbiota from wild-type mice did not change Aβ level. Gut microbiota plays an important role in controlling astrocyte activation, morphology, and recruitment to Aβ plaques [141]. It also regulates blood-cerebrospinal fluid barrier function and Aβ pathology [142]. Additionally, intravenous injection of outer membrane vesicles derived from AD patients into healthy mice for eight weeks increases the BBB permeability with elevated levels of brain inflammatory markers, glial cell activation, tau hyperphosphorylation, and cognitive impairment in the recipient mice [143]. These findings suggest that gut microbiota can influence brain function and lead to AD-like pathologies and cognitive deficits through the microbiota-gut-brain axis.

## Role of glial cells in AD

### Role of microglia in AD

Activated microglia are found surrounding senile plaques in the brains of AD patients [144]. Studies have revealed that microglia rapidly accumulate around newly formed Aβ plaques, and express various receptors involved in Aβ binding and phagocytosis, such as scavenger receptor A1, CD36 [145], CD14, TLR2, TLR4, TLR6, and TLR9 [146]. This suggests that microglia play a role in the clearance of Aβ. However, microglia isolated from the brains of AD mice exhibit decreased ability to phagocytose Aβ, possibly due to long exposure to an Aβ-rich environment that has impaired their phagocytic function [147]. Additionally, among the numerous AD risk genes reported, myeloid cell trigger receptor 2 (*TREM2*) and *CD33* primarily act through microglia, and genetic variations in both genes can lead to reduced uptake and clearance of Aβ by microglia [147]. TREM2, CD33, and CD22 can influence the intracellular adaptor molecule CARD9; the latter can attenuate Aβ pathology and modify microglial responses in AD mice [148, 149]. Obesity also affects the function of central microglia through peripheral inflammatory responses [150]. During aging, the function of BBB is diminished, allowing peripheral inflammatory factors to enter the brain and activate microglia.

Microglia can exhibit two different phenotypes, M1 and M2, in response to different stages of inflammation or various stimulating factors [151]. M1 microglia release pro-inflammatory cytokines and exhibit reduced phagocytic capacity, while M2 microglia release anti-inflammatory cytokines and have enhanced phagocytic ability. However, the activated microglia can have beneficial or detrimental effects depending on the brain region observed, disease stage, disease model, and other factors [152]. In normal individuals, microglial activation occurs in response to neuronal damage, abnormal protein folding or aggregation, leading to the production of immune-inflammatory reactions. Optogenetic stimulation of microglia can efficiently promote both Aβ clearance and synaptic elimination in the brain parenchyma, while inhibiting C1q selectively prevents synaptic loss induced by microglial depolarization without affecting Aβ clearance [153].

Once damaged neurons or abnormal proteins are phagocytosed and cleared, inflammation subsides, and local homeostasis is restored. In the process of AD, continuous accumulation of pathologies, such as Aβ, leads to persistent microglial activation [152]. The chronic activation of microglia results in a prolonged and unresolved inflammatory state, contributing to the progression and pathogenesis of AD. Activated microglia release a large number of cytokines such as TNF-α, IL-6, IL-1α, NO, and C1q, which can directly damage the neurons. Activated microglia can engulf synapses, leading to impaired learning and memory. Dysfunctional microglia can also induce transformation of astrocytes to a toxic A1 phenotype and thereby decrease nutritional support to neurons [152]. Microglia are the major cell type expressing complement C3a receptor (C3aR) in the brain. Depletion of C3aR can reverse the HIF-1α-induced metabolic impairment and enhance microglial response to Aβ pathology [154].

### Role of astrocytes in AD

Astrocytes are the most abundant cell population in the human brain and play essential roles in supporting, nourishing, and protecting neurons through various functions such as regulating neurotransmitter release and reuptake, energy metabolism, signaling pathways, ion buffering, and blood flow regulation [155]. Activation of astrocytes is involved in both neural repair and toxicity [156]. The gene expression profiling data from stimulated astrocytes reveal two forms of astrocyte activation: A1 phenotype, induced by LPS or neuronal injury, and A2 phenotype, induced by ischemia. A1 astrocytes predominantly express genes associated with the classical complement pathway, with reduced phagocytic capacity and diminished ability to promote synapse growth. They also produce neurotoxic substances that contribute to neuronal death. A2 astrocytes mainly express neurotrophic factors and possess reparative properties [157]. It is still not fully understood which factors control this phenotype transformation of astrocytes, and whether and how it is applicable for AD.

Astrocyte activation is an early event in AD and can occur prior to Aβ deposition. Astrocyte reactivity, as an important upstream event linking Aβ with initial tau pathology, may have implications for the biological definition of preclinical AD and cognitively unimpaired individuals for clinical trials [158]. Primary astrocytes treated with Aβ oligomers or astrocytes in the brains of APP/PS1 mice show morphological changes with increased level of GFAP, a marker for astrocyte activation. In susceptible brain regions of AD patients, such as the frontal lobe and hippocampus, there is a significant increase in astrocytes expressing A1-type markers such as complement C3, compared to normal controls, suggesting A1-type astrocyte activation. Activated astrocytes can also release cytokines, interleukins, and NO, exacerbating inflammatory reactions and causing damage to neurons. At the same time, astrocytes can internalize and degrade Aβ, a process that requires APOE. Astrocytes exposed to Aβ deposits show upregulated expression of extracellular Aβ-degrading enzymes such as neprilysin and IDE. Exercise-induced irisin significantly reduces Aβ pathology by increasing astrocytic release of the Aβ-degrading enzyme neprilysin [159].

In addition, astrocytic endfeet form an important component of the BBB, and the perivascular space between endothelial cells and astrocytic endfeet serves as a pathway for brain glymphatic circulation. Knockout of aquaporin-4, a water channel protein expressed in astrocytes, leads to impairment of brain glymphatic clearance and increased Aβ deposition [160]. Therefore, astrocyte dysfunction can also impair the clearance of brain metabolites and facilitate abnormal protein aggregation through damaging the glymphatic clearance system. Additionally, NOX2, Toll-like receptors, and the nuclear factor kappa-B (NF-κB) pathway activation also play important roles in AD.

## Role of Aβ in AD neurodegeneration

### Mechanisms underlying Aβ generation and degradation

Amyloid pathology is one of the major AD pathologies. It is generally believed that the amyloid pathology occurs preceding tau pathology. In FAD, amyloid accumulation is a hallmark of early AD development and/or a triggering event, whereas tau pathology generally comes later with more solid link to cognition/behavior issues.

#### Aβ generation pathways

Aβ is produced from APP. Located on chromosome 21 and spanning approximately 190 kb pairs, the *APP* gene consists of at least 18 exons. By alternative splicing, at least 10 different mRNA isoforms are produced, directing the translation of protein isoforms ranging from 365 to 770 amino acid residues [161]. The human brain predominantly expresses APP695 and APP770 [162]. As a type I transmembrane protein, APP comprises a long extracellular N-terminal segment and a short intracellular C-terminal segment. By interacting with the extracellular matrix, APP participates in the regulation of neuronal plasticity and repair of damaged tissues [163].

(1) Amyloidogenic pathway

β-Secretase, referred to as β-site APP-cleaving enzyme-1 (BACE1), cleaves APP695 at Asp1 between Met596 and Asp597, resulting in the release of 99-aa residue membrane-associated C-terminal fragment (CTF or C99) [164–166]. The C99 is then cleaved by γ-secretase to produce full-length Aβ composed of 39–43 amino acids including the N-terminal 28 amino acids of APP transmembrane region and an adjacent 11–15 amino acids in the transmembrane region [167]. Due to the hydrophobic nature of the last few amino acid residues at the C-terminus, the Aβ peptides with longer C-terminal are more prone to aggregation and deposition. Among different forms of Aβ, Aβ1-40 and Aβ1-42 are the most extensively investigated Aβ forms in AD research.

Recent studies show that asparagine endopeptidase (AEP) can cut APP at N373 or N585 on the extracellular space to facilitate Aβ production. The molecular mechanisms may involve removal of the N-terminal domain on APP, which facilitates more efficient BACE1 cleavage of the resultant APP C596–695 fragment. This AEP-mediated APP cleavage is also termed as the δ-secretase pathway [168–170]. AEP can also directly cut BACE1 at N294, which enhances BACE1 activity and shifts the optimal pH of BACE1 from acidic to neutral, so that BACE1 could process APP even under neutral pH or at extracellular compartment [169].

**Fig. 1:**
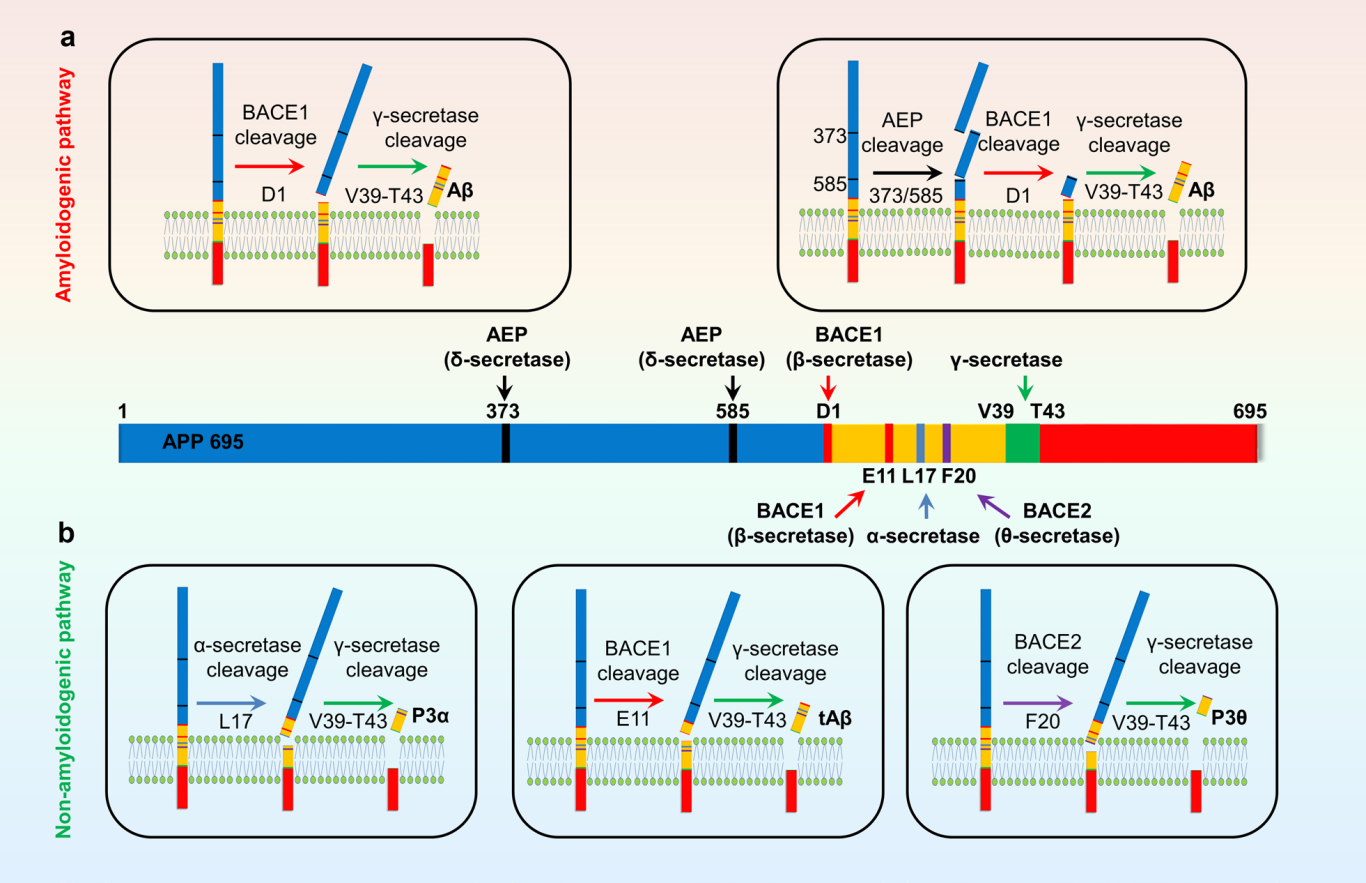
Amyloidogenic and non-amyloidogenic APP processing pathways. **a** The amyloidogenic processing pathway of APP produces full-length Aβ through BACE1 and γ-secretase cleavage. AEP cleavage at N373 and N585 makes APP more susceptible to BACE1 and thus promotes Aβ production. **b** The non-amyloidogenic processing pathway of APP by α-secretase within the Aβ domain or by BACE1 at Glu11 or by BACE2 at Phe20 does not produce full-length Aβ

β-Secretase cleavage of APP mainly takes place in endosomes and lysosomes. Both APP and BACE1 are type I transmembrane proteins that are initially inserted into the cell membrane and then undergo internalization into endosomes and further fuse with early lysosomes. The acidic environment within endosomes and lysosomes facilitates the cleavage of APP by BACE1. BACE1 is enriched in lipid raft, which is the potential subcellular localization for Aβ generation. Active γ-secretase has been detected in cell membrane, endosomes, and lysosomes [171] (Fig. 1).

(2) Non-amyloidogenic pathway

In physiological conditions, a majority of APP is processed by α-secretase which involves the cleavage of the peptide bond between Lys16 and Leu17 of Aβ, resulting in the production of a larger, N-terminal, soluble sAPPα fragment (sAPPα) and a C-terminal fragment of 83 amino acids (CTFα or C83) [172]. The sAPPα is secreted into the extracellular space, while C83 remains membrane-bound, which is further cleaved by γ-secretase generating a P3α fragment and CTFγ. It is generally believed that α-secretase cleavage of APP occurs at the cell membrane, which does not generate complete Aβ molecules. In addition, APP can be cleaved by BACE2 (namely θ-secretase) at Phe20 within the Aβ domain, leading to the formation of CTFθ (or C80). C80 is further cleaved by γ-secretase, generating a P3θ fragment and CTFγ that also does not produce Aβ [173–175]. BACE1 can also cleave APP at Glu11 within Aβ region, which produces C89 and a truncated Aβ11-40/42 (tAβ) [164, 166]. Figure 1 summarizes the amyloidogenic and non-amyloidogenic pathways for APP processing and Aβ production.

#### Aβ degradation and clearance

Normally, Aβ generation is counterbalanced by the proteolytic degradation. The involved enzymes for Aβ degradation and clearance include zinc metalloproteinase neprilysin [176], the most efficient Aβ peptidase located in the intraluminal/extracellular space and the early Golgi and ER compartments; the membrane-bound endothelin converting enzymes 1 and 2; the intracellular insulin-degrading enzyme (IDE) [177]; and plasmin, a serine protease that can degrade both monomer and fibril Aβ [178]. In addition, the matrix metalloproteases (MMPs) MMP2 and MMP9 can degrade Aβ in vitro. Cathepsin D, an aspartyl protease localized within lysosomes and endosomes, is a major Aβ-degrading enzyme in brain homogenates.

Aβ is also cleared through cell-mediated mechanisms, such as phagocytosis by microglial cells [179], transport from brain tissue to the periphery via binding with lipoproteins and mediated by related transporters such as LRP and VLDL-R [180, 181]. Capillary dysfunction impedes Aβ clearance [182]. Age-dependent loss of myelin integrity can be a driver or a risk factor of Aβ deposition [183]. β2-microglobulin (β2M), a component of major histocompatibility complex class I (MHC class I), is upregulated in AD brains and constitutes the core of Aβ plaque. A recent study indicates coaggregation of β2M with Aβ, which contributes to cognitive deficits in AD model mice [184]. Studies also show that the lymphatic system in the brain can accelerate Aβ clearance during deep sleep [185]. Thus, sleep disorders can lead to reduced Aβ clearance in the brain and promote neurodegeneration [186]. APOE4 synergizes with sleep disruption to accelerate Aβ deposition and Aβ-associated tau seeding and spreading [187].

Together, impaired degradation and/or clearance of Aβ may be caused by dysfunction of specific proteolysis or clearance systems, with a subsequent consequence of Aβ aggregation and deposition during the long course of AD.

### Mechanisms underlying Aβ overproduction

Several factors, such as gene mutations in *APP* and presenilin (PS) catalytic subunit (γ-secretase), post-translational modifications, *APOE4*, aging and various environmental stimuli, can contribute to Aβ overproduction and aggregation in FAD or sporadic AD patients. However, the detailed mechanisms remain largely unclear.

#### *APP* gene mutations and post-translational modifications

Several *APP* mutations have been identified in FAD patients, and these mutations directly affect Aβ generation. The Swedish and E674Q mutations alter APP structure, making it more susceptible to the BACE1 cleavage [188, 189]. The Arctic and Dutch mutations occur within the Aβ peptide, making it more prone to aggregation [190]. Austrian, Iranian, French, German, and other mutations located at the C-terminal of APP promote the production of longer Aβ fragments [191]. The Flemish mutation, located in the substrate inhibitory domain of APP, results in increased APP cleavage by γ-secretase [192]. K16E or K16F mutation in Aβ1-28 and Aβ25-35 fragments leads to greater susceptibility to aggregation, and Zn^2+^-binding makes Aβ more stable [193].

During the constitutive secretory pathway, APP undergoes extensive post-translational modifications, including glycosylation, phosphorylation, sulfation, palmitoylation, ubiquitination and SUMOylation. Among them, increased Thr668 phosphorylation of APP has been extensively detected in AD brains with mechanisms involving increased DYRK1A (dual-specificity tyrosine(Y)-phosphorylation regulated kinase 1A) [194]. It is generally recognized that Thr668 phosphorylation facilitates β- and γ-cleavages and increases Aβ generation, although opposite results were also reported. In addition, phosphorylated Tyr682 and Tyr687 have been exclusively detected in AD brains but not in healthy controls, and these two sites seem to negatively regulate Aβ generation [195].

#### Activation of β-secretase

β-secretase (BACE1) is widely expressed in neurons, oligodendrocytes and astrocytes. It is predominantly localized in the acidic intracellular compartments (such as late Golgi/TGN and endosomes) with an optimal enzymatic activity at pH 4.5. The mRNA expression and activity of BACE1 are increased in the brain, CSF, peripheral blood mononuclear cells and plasma of the elderly as well as probable AD and AD patients, suggesting that plasma BACE1 activity may serve as a biomarker for predicting AD [196, 197].

Epigenetic modulations, including DNA methylation, non-coding RNA alterations, and histone modifications, are of great significance in regulating Aβ metabolism. For instance, chromatin remodeling assists BACE1 upregulation and Aβ production [196, 198]. A global decrease of DNA methylation has been detected in the hippocampus of AD patients [199], and histone hyperacetylation and DNA hypomethylation can increase APP and BACE1 transcription, possibly by activating NF-κB [200]. APP and BACE1 are upregulated as a result of demethylation at their promoters, and S-adenosylhomocysteine treatment induces hypomethylation of *PSEN1* and *APP* accompanied by their overexpression and Aβ overproduction [201]. In addition, various types of post-translational modifications of BACE1 at multiple sites have been reported to play a crucial role in BACE1 trafficking and maturation and thus contribute to Aβ overproduction and aggregation. These modifications include acetylation at Lys-126, Lys-275, Lys-279, Lys-285, Lys-299, Lys-300, or Lys-307; N-glycosylation at Asp153, Asp172, Asp223 or Asp354; palmitoylation at Cys474, Cys478, Cys482, or Cys485; phosphorylation at Ser498 or Thr252; ubiquitination at Lys203, Lys382, or Lys501; and SUMOylation at Lys275 or Lys501 [202, 203].

#### Abnormalities of γ-secretase

γ-Secretase is a complex composed of PS1 (467 aa), PS2 (488 aa), Nicastrin (~ 130 kDa), APH-1 (30 kDa), and PEN-2 (12 kDa) [204], in which PS1 and PS2 can directly cleave APP at at least five adjacent sites and thus produce Aβ with 39 to 43 amino acid residuals, most commonly Aβ42. PS1 and PS2 are highly homologous 8-transmembrane proteins with 10 hydrophobic regions inserted in the membrane [205, 206], with hydrophilic N- and C-terminal located in the cytoplasm. Nicastrin is a glycoprotein and its maturation depends on the PS-mediated transport from ER to the cell membrane. Nicastrin, APH-1 and PEN-2 in the complex can stabilize or regulate PS and thus participate in γ-secretase cleavage [207–209]. A recent study shows that ganglioside GM1, the most common brain ganglioside, can specifically accelerate γ-secretase cleavage of APP without affecting other substrates including Notch1, potentially through its interaction with the N-terminal fragment of PS1 [210].

PS may be an aspartic acid-dependent protein hydrolase. Inhibiting γ-secretase can reduce the intracellular Aβ level [211]. To date, more than 400 mutations in *PSEN* and *APP* genes have been identified in early-onset FAD, with *PSEN1* and *PSEN2* mutations accounting for ~ 75% and ~ 12%, respectively [212]. FAD patients with *PSEN* mutations exhibit elevated Aβ levels in plasma and the brain. Both in vitro and in vivo experiments have confirmed that almost all *PSEN* mutations ultimately result in increased production of longer Aβ fragments and an elevated Aβ42/Aβ40 ratio. *PSEN* gene mutations may promote Aβ toxicity by simultaneously affecting APP cleavage, endocytosis, transport, and functional abnormalities, such as ER calcium homeostasis, autophagy pathways, and neuronal endocytosis.

#### Upregulation of δ-secretase

Recent studies reveal that AEP as a δ-secretase can cut APP to facilitate Aβ production. AEP is a cysteine protease that specifically hydrolyzes peptide bonds after asparagine residues in mammals. It has been observed that AEP is activated in normal mice in an age-dependent manner, and it is strongly activated in 5 × FAD transgenic mice and in human AD brains. Activation of AEP drives the onset of AD through cleaving tau and APP. The AEP-mediated cleavage of these peptides enhances amyloidosis and tau hyperphosphorylation, and thus induces neurodegeneration and cognitive impairment [168, 213].

As mentioned above, α-secretase cleavage of APP predominantly occurs at the plasma membrane that does not produce Aβ. Three members of the α-disintegrin and metalloproteinase (ADAM) family, ADAM9, ADAM10 and ADAM17, have been identified to possess α-secretase-like activity, which is regulated by multiple factors such as protein kinase C in the trans-Golgi-network [214]. However, it is currently not clear whether and how α-secretase plays participates in AD [215].

### Mechanisms underlying Aβ aggregation

Aβ monomers can form higher-order assemblies ranging from low-molecular-weight oligomers (including dimers, trimers, tetramers, and pentamers) to midrange-molecular-weight oligomers, high-molecular-weight oligomers, protofibrils, fibrils and senile plaques. Soluble Aβ can interact with potential receptors and activate downstream pathways to generate reactive oxygen species, tau hyperphosphorylation and inflammatory responses [216]. The extracellular accumulation of insoluble Aβ can also activate neurotoxic cascades that ultimately lead to cytoskeletal changes, neuronal dysfunction and neural death [217, 218].

Compared with Aβ production, much less has been clarified for Aβ aggregation. Following production, Aβ interacts with receptors for advanced glycation end products (RAGE), which facilitates the transportation of Aβ across the BBB [219], leading to Aβ accumulation within the brain. RAGE also stimulates BACE1 expression through generating an intracellular Ca^2+^ response that activates NFAT1 (nuclear factor of activated T-cells 1), an activator of BACE1. BACE1 then cleaves APP to produce Aβ, forming a feedback loop to aggravate Aβ accumulation [220].

Impaired Aβ clearance could also promote Aβ accumulation. Several proteinase inhibitors, such as α1-antichymotrypsin and nexin-1, have been detected in the senile plaques of AD patients, which prevents the timely clearance of Aβ by proteases and lead to Aβ deposition [221, 222]. Inhibition of Aβ-degrading enzymes, such as neprilysin and IDE, can also result in Aβ accumulation.

Increased neuronal activity also promotes Aβ production and release [223]. Patients with temporal epilepsy often exhibit Aβ deposition in the brain at as early as 30 years of age. The frontal, parietal, and posterior cingulate cortices are the most vulnerable brain regions for Aβ deposition in AD patients, and these brain areas also show highest neuronal metabolic activity [223–225]. Several physicochemical factors, such as aluminum, iron, zinc, and acidic pH (pH 4–7), also promote Aβ aggregation.

### Mechanisms or pathways involved in Aβ toxicity

Aβ is the main component of amyloid-like plaques in the brains of AD patients. Synthetic Aβ peptides can exert toxic effects both in vitro and in vivo. Down syndrome patients with trisomy 21 (owning triplicate *APP* gene) exhibit typical AD-like neuropathological changes and clinical manifestations, while those with duplicate chromosome 21 do not exhibit AD-like changes even at an old age [226, 227]. The FAD patients carrying mutations in *APP* or *PSEN* have increased Aβ or elevated Aβ42/Aβ40 ratio [228], and show earlier development of dementia and more rapid disease progression [229]. Several lines of transgenic mice carrying human mutant *APP* gene exhibit age-dependent increases of extracellular Aβ level and develop neuropathological and behavioral changes resembling AD [230]. Homozygosity for *APOE*4 increases Aβ burden in human brains [68].

Despite these supporting evidence, the role of Aβ in the pathogenesis of AD remains controversial. For instance, the relationship between Aβ level or brain amyloid plaque burden and the severity of cognitive impairment remains unclear; drugs targeting Aβ or its metabolism have not yet achieved the expected therapeutic effects in clinical trials, though inspiring progress has been made most recently. Therefore, Aβ may be a necessary factor but not sufficient for the development of AD. The toxicity of Aβ may require the involvement or synergistic action of other pathogenic molecules, such as tau.

#### Aβ induces oxidative damage

AD patients show elevated activities of superoxide dismutase and glucose-6-phosphate dehydrogenase, decreased activity of glutamine synthetase, and increased lipid peroxidation, indicating a close relationship between free radicals/oxidative damage and AD. Possible pathways through which Aβ induces oxidative damage in neuronal cells are as follows.

Aβ can induce the production of free radicals, causing extensive and severe damage to the cell membrane. Aβ primarily targets the phospholipid bilayer structure of the plasma membrane, specifically polyunsaturated fatty acids with > C = C < double bonds, leading to the formation of cytotoxic lipid radicals and lipid peroxides through their reaction with free radicals [231]. The lipid peroxides can be further decomposed to generate more free radicals, which act on other double bonds, resulting in a chain reaction of free radicals. Metal ions such as iron [232] and copper [233] and their complexes can destruct cell membranes, and increase membrane fluidity and permeability, tissue edema, and necrosis. Recent studies show that atomic structures assembled by Aβ can disrupt neuronal cell membrane, allowing water and ions to pass through, ultimately leading to cell swelling and death.

Increased Aβ causes damage to mitochondria through disruption of intracellular calcium homeostasis. Aβ can form channels in the lipid bilayer of cell membrane, which allows Ca^2+^ influx, leading to intracellular calcium overload and oxidative stress [234–236]. The increased calcium mediates phospholipase activation, which leads to an elevation in arachidonic acid levels, ultimately resulting in increased generation of oxygen free radicals. Mitochondrial calcium overload suppresses mitochondrial membrane potential and thus increases the level of superoxide anions. Calcium channel blockers can alleviate the cytotoxicity of Aβ [237, 238]. Amyloid-binding alcohol dehydrogenase (ABAD), also known as endoplasmic reticulum (ER) amyloid β-peptide binding protein, is composed of 262 amino acids and mainly present in the liver and the heart. In normal conditions, it is expressed at a low level in neurons. In AD brains, especially in the vicinity of Aβ deposits, ABAD is significantly increased. ABAD itself lacks a signal peptide and a transmembrane domain. Binding to Aβ42 initiates its translocation from ER to the plasma membrane; and ABAD directly links Aβ to mitochondrial toxicity in AD [239, 240]. The formation of the ABAD-Aβ complexes during this process has a toxic effect on neurons. The binding of Aβ42 to ABAD also affects the transport of APP, leading to the retention of APP, tau, α-synuclein, and other proteins in the ER, thereby impairing neuronal function [241]. Targeting the Aβ-ABAD interaction is emerging as a novel therapeutic strategy for AD [242, 243].

Reactive astrocytes surrounding senile plaques are one of the pathological hallmarks of AD, and astrocytes play important roles in the uptake of extracellular glutamate [244]. In cultured astrocytes, free radicals induced by Aβ can inhibit glutamate uptake, resulting in an increased extracellular glutamate level and excitotoxicity to neurons. As the astrocytic uptake of glutamate is ATP-dependent, impairment in glucose metabolism can suppress glutamate uptake. In addition, protein oxidation increases carbonyl content at histidine, proline, arginine, and lysine. These changes can lead to inactivation of some key enzymes, such as glutamine synthetase and creatine kinase [245].

#### Aβ induces inflammatory response

Various complement components (including C1q, C4d, C3b, C3C, C3d, and C5b-9), acute-phase proteins, inflammatory markers, and activated glial cells are found within or around the senile plaques of AD patients. Aβ stimulates astrocytes to produce excessive complement C3 [246]. Aβ can bind with C1q and activate the non-antibody-dependent classical complement pathway [247]. NLRP3 is a key molecule involved in inflammasome activation, and knocking out NLRP3 can reverse cognitive impairments in APP/PS1 mice [248]. The level of cleaved caspase-1, a marker of inflammasome activation, is significantly increased in AD brains. Application of non-steroidal anti-inflammatory drugs can delay or prevent AD. These lines of evidence suggest that the toxic effects of Aβ involve inflammatory processes.

Microglia play an indispensable role in mediating Aβ toxicity. Treatment of cultured neurons with 100 μM Aβ (approximately 1000 times the physiological level) does not cause significant neuronal damage. However, when neurons were cultured with microglia, only 100 nM Aβ treatment exhibits significant toxic effects.

#### Aβ induces synaptic dysfunction

Synaptic damage is an early event in AD neurodegeneration. Studies suggest that soluble Aβ may have stronger and earlier detrimental effects on synapses than deposited Aβ [249]. Soluble Aβ refers to the Aβ that remains in the aqueous solution after brain tissue extraction and high-speed centrifugation, and monomeric form (4 kDa) and oligomeric forms (approximately 8 kDa and 12 kDa) have been detected using ELISA and Western blotting [250]. Artificially synthesized Aβ-derived diffusible ligands (ADDLs) are small spherical structures with a diameter of approximately 5 nm, generated by incubating synthetic Aβ1-42 in cold Ham's F12 medium. In SDS-PAGE, these ADDLs exhibit apparent molecular weights of approximately 4, 8, 16, and 18 kDa. Low-molecular-weight ADDLs tend to be located at the postsynaptic sites and may induce microglial phagocytosis of synapses by recruiting activated complement factors such as C3 and C1q, resulting in dendritic spine loss and synaptic damage [86, 251]. A recent study investigated the effects of Aβ phosphorylation on neuronal autophagy and the endo-lysosomal pathway. They found that Ser8-phosphorylated Aβ accumulates in autophagosomes, while the Ser26-phosphorylated Aβ is located to lysosomes. The selective sorting of phosphorylated Aβ species results in differential impairment of vesicular transport and lysosomal function, contributing to neurotoxicity [252].

Aβ exerts toxic effects through direct or indirect interactions with various receptors, including glutamate receptors (AMPA receptor, NMDA receptors [NMDARs], metabotropic glutamate receptor 5 [mGluR5]), cholinergic receptor α7-nAChR, insulin receptor, neurotrophin receptor P75NTR, RAGE, Ephrins receptors EphB2 and EphA4, and prion protein PrP [253]. Aβ can activate the metabotropic glutamate receptor mGluR5, leading to activation of protein kinases such as p38-MAPK, JNK, and cyclin-dependent kinase (Cdk) 5, resulting in tau hyperphosphorylation and impairment of long-term potentiation (LTP) [254]. Aβ can also activate NMDAR and PP2B via calcium-mediated signaling, leading to nuclear translocation of NFATc4 (nuclear factor of activated T-cells) and loss of dendritic spines. Aβ oligomer can upregulate α7-nAChR, by which it inhibits extracellular signal-related protein kinase 2 (ERK2), and subsequently suppress cAMP-response element binding protein (CREB) phosphorylation and downregulate BDNF, and finally results in impairment of LTP [255]. Interaction of Aβ with RAGE and scavenger receptor leads to neurodegeneration and death. A recent study identified synaptic binding of transmembrane protein 97 with Aβ in the human AD brain, which might be involved in the synapse-associated toxicity of Aβ [256].

#### Aβ induces impairment of neuronal axoplasmic transport

**Fig. 2:**
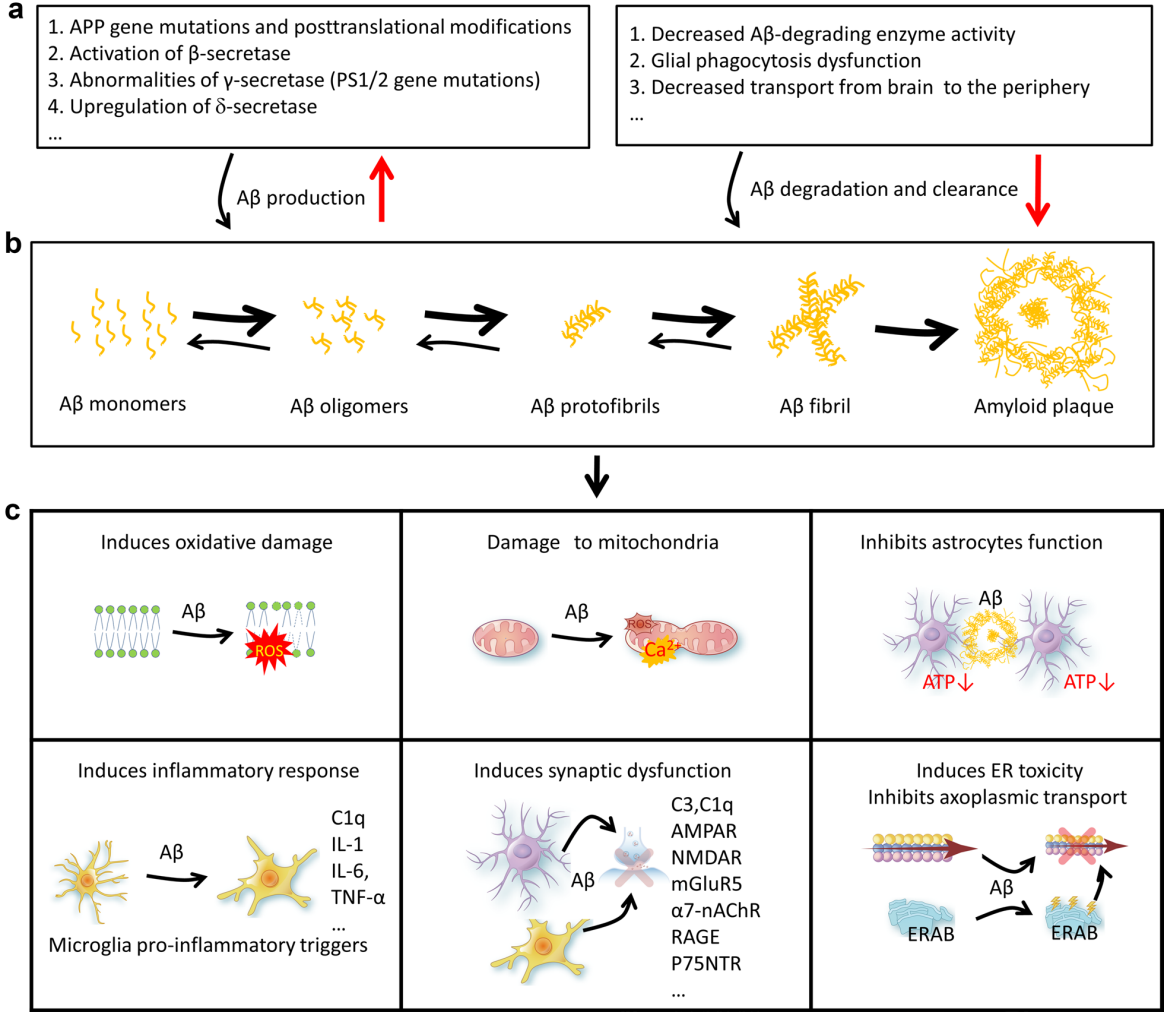
Mechanisms underlying Aβ accumulation and toxicities. **a** Common factors that promote Aβ production (left) or contribute to Aβ accumulation (right). **b** Experimentally proven processes of amyloid plaque formation. **c** Neural toxicities of Aβ

After synthesis in the ER of neurons, APP is initially transported through axons to synaptic terminals, and then undergoes intracellular transcytosis to return to the neuronal cell body and dendrites. This transport relies on the interaction between APP and PS, and plays a crucial role in maintaining normal APP metabolism and affects the generation of Aβ [257, 258]. In FAD, mutations in either *APP* or *PSEN* gene can disrupt interactions between APP and PS, leading to impaired APP transport and Aβ overproduction [259]. In sporadic AD, the overall level of Aβ may be not significantly elevated, but the following factors may lead to localized Aβ aggregation affecting APP transport: (1) free radicals covalently bind to Aβ, forming localized nuclei or seed crystals which aggregate within cells and inhibit APP transport; (2) positively charged proteins such as heparan sulfate proteoglycans can accelerate Aβ aggregation; and (3) specific intracellular locations of serum amyloid component P, composed of two identical pentamers with each molecule having 10 Aβ-binding sites, can lead to local Aβ aggregation [260]. Together, Aβ and tau accumulation are the recognized pathologies in AD. Their interplay can drive AD progression through complex mechanisms. Figure 2 summarizes the mechanisms underlying Aβ accumulation and the toxicities.

## Role of tau in AD neurodegeneration

Accumulation of hyperphosphorylated tau forming NFTs is a hallmark of AD. Tau pathology is positively correlated with cognitive decline. In this part, we will review how tau proteins become hyperphosphorylated and accumulated, and how the chronically accumulated tau induces neurodegeneration.

### Biology of tau proteins

**Fig. 3:**
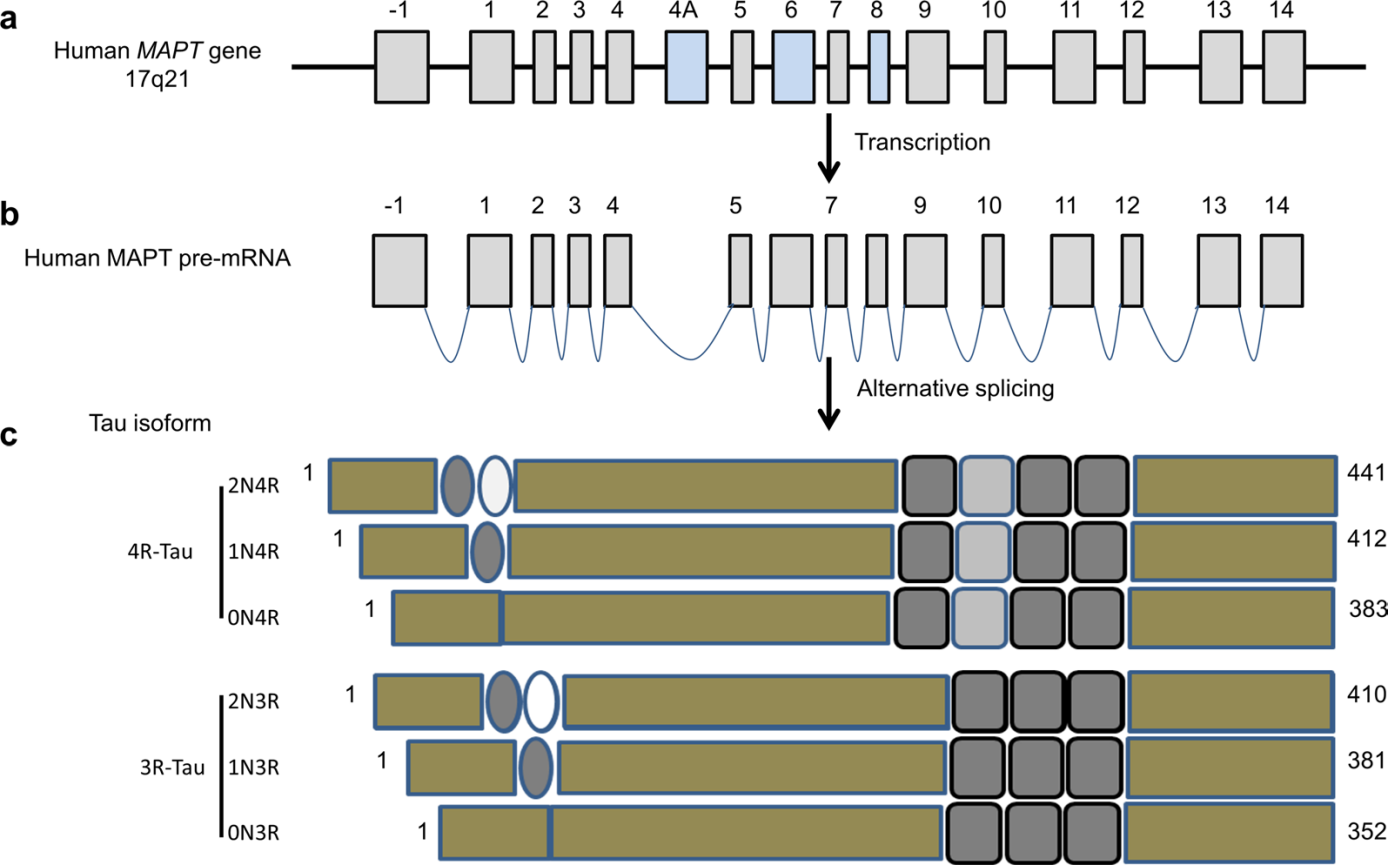
Schematics showing basic structures of human tau. **a** *MAPT* gene, **b** pre-mRNA after transcription, and **c** the six major protein isoforms produced by alternative splicing

As a cytoskeleton component, tau accounts for over 80% of microtubule-binding proteins in neuronal cells. The classical function of tau is to promote microtubule assembly and maintain the stability of microtubules [261]. In SDS-PAGE, tau isolated from normal adult human brains shows at least six isoforms with an apparent molecular weight of approximately 48 kDa to 60 kDa [262]. These isoforms are various splicing products (352–441 amino acid residues) derived from a single gene (*MAPT*) located on chromosome 17 [263]. According to the numbers of N-terminal inserts and C-terminal microtubule-binding repeats, the tau proteins are classified into 0N-3R-tau (or 0N-4R-tau), 1N-3R-tau (or 1N-4R-tau), and 2N-3R-tau (or 2N-4R-tau), containing 0, 1, or 2 N-terminal inserts (0 or 29 or 58 aa), and 3 or 4 C-terminal microtubule-binding repeats (31–32 aa each) [264]. In fetal brains, only 0N-3R-tau at ~ 48 kDa has been detected by SDS-PAGE (Fig. 3) [265].

**Fig. 4:**
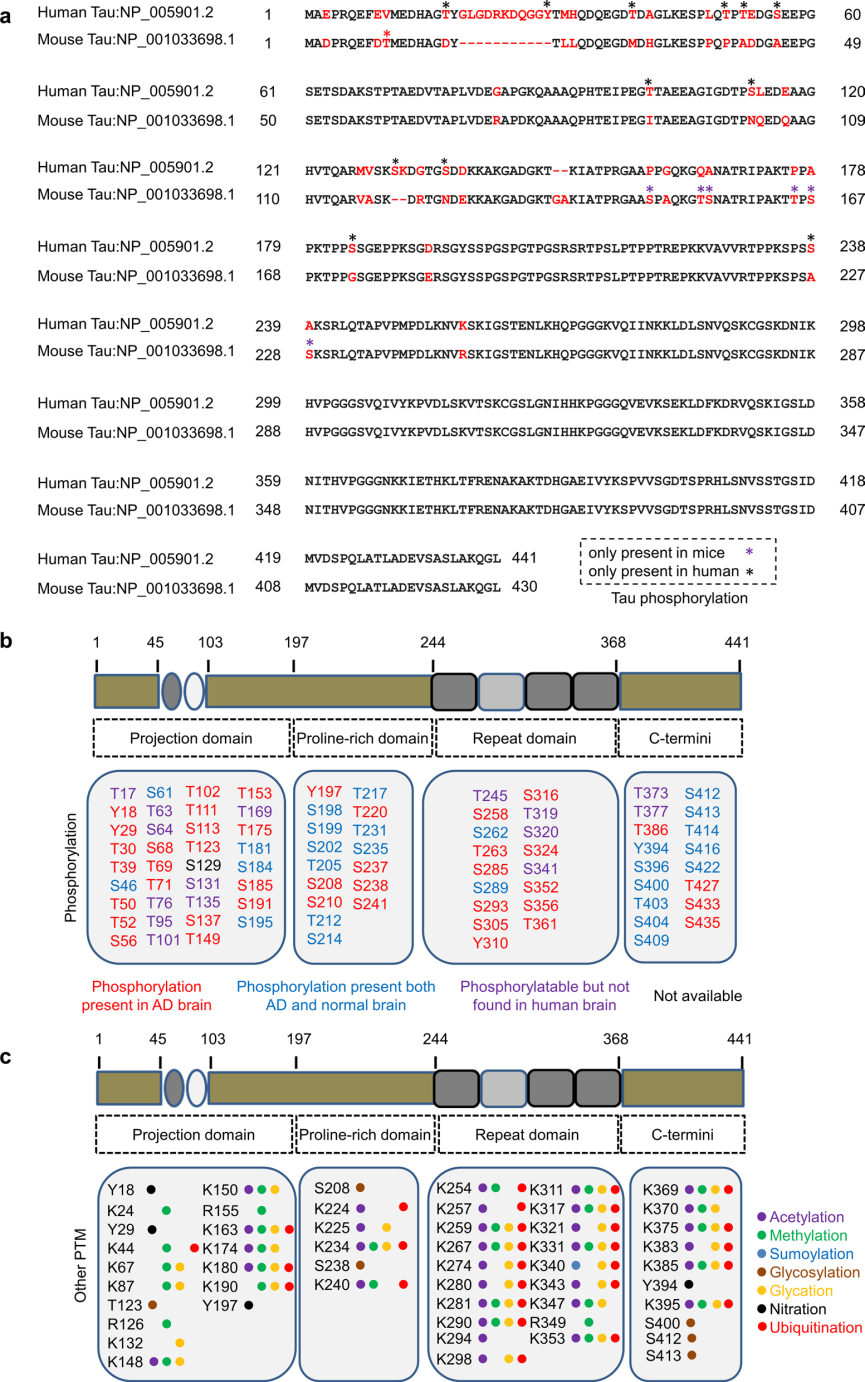
Protein sequences and post-translational modifications of human and mouse tau proteins. **a** Sequence alignment of human tau (NP_005901.2) and mouse tau (NP_001033698.1). Red stars, potential phosphorylation sites only in mouse tau; black stars, potential phosphorylation sites only in human tau. The inconsistent amino acids between murine Tau430 and human Tau441 are labeled in red. **b** Tau phosphorylation site: red represents phosphorylation sites found exclusively in the brains of AD patients; green indicates phosphorylation sites found exclusively in non-AD human brains; blue represents the phosphorylation sites found in both non-AD and AD patients; and black represents no-phosphorylation detected. **c** Other identified post-translational modifications of tau proteins

The full-length tau share 89% amino acid homology between humans (441aa or Tau441) and mice (430 aa), with the main differences located in the N-terminal projection segment [266]. The full-length human tau contains 85 potential phosphorylation sites (80 serine/threonine sites and 5 tyrosine sites), and over 60 sites have been detected in AD or non-AD brains. Some of the phosphorylation sites are within the four-repeat (4R) domains (spanning from T245 to V363): S258, S262 and T263 are located inside R1; S289 and S293 in R2; S305, Y310 and S316 in R3, and S352, S356 and T361 in R4 [267–270]. Regarding the difference of phosphorylation sites between human and rodent tau, T17, T39, T50, T52, T101, S56, S113, S131, S137, S184, S238, and Y29 are the potential human phosphorylation sites not found in mouse tau, while T10, T154, T165, S148, S155, S167, and S228 are the potential mouse sites not found in human tau [266]. In addition to phosphorylation, other post-translational modifications have also been reported in tau (Fig. 4).

Biochemical analyses show that in normal brains tau has a phosphate content ranging from 2 to 3 moles per mole of tau, while the phosphate content increases to 5–9 moles per mole of tau in the brains of AD patients [271]. By SDS-PAGE, three major bands with apparent molecular weights of approximately 62–72 kDa are shown in the brain extracts of AD patients [272]. Tau proteins in AD brains can be divided into three fractions: cytoplasmic normal tau (C-tau), abnormally modified soluble tau (AD P-tau), and tau proteins abnormally modified and aggregated into paired helical filaments (PHF-tau) [273]. Under electron microscope, PHFs are shown as a right-handed helical coil with a diameter of approximately 22–24 nm, and a narrow region of ~ 10 nm at intervals of every 80 nm [274]. Abnormalities in tau protein have been observed in a class of neurodegenerative diseases known as tauopathies, including AD. Except frontotemporal dementia with Parkinsonism linked to chromosome 17 (FTDP-17), which is caused by *MAPT* mutations, the remaining tauopathies are associated with abnormal post-translational modifications of tau [275] (Fig. 4).

Tau protein was first discovered as a microtubule-associated protein in 1970s [276]. Since then, studies on tau had been mainly focused on its function as a cytoskeleton protein. Recently, the non-cytoskeletal roles of tau have received increasing attention. Many new binding partners for tau have been identified, including DNA, RNA, RNA-binding proteins, transcription factors, and some membrane receptors [277–281]. The novel functions of tau, such as the roles in chromosomal stability, gene expression, aprotein synthesis and cell viability, have been proposed based on the diversity of tau-binding partners coupled with the discovery of tau in the nuclei and synapses. The tau–tau interaction and propagation have led to the hypothesis that a prion-like function of pTau may be central to tauopathies [282].

### Distinct cellular effects of tau phosphorylation and aggregation

Tau protein in AD brains undergoes abnormal phosphorylation, aberrant glycosylation, glycation, ubiquitination, nitration, acetylation, SUMOylation, and abnormal truncation, etc. [268]. Among these post-translational modifications, the role of tau hyperphosphorylation in AD neurodegeneration has been most extensively studied.

#### Responsive or reactive tau phosphorylation endows cell resistance to apoptosis

As pTau is the main component of NFTs in degenerating neurons of AD patients, many scientists assume that phosphorylation of tau may promote neuronal apoptosis. However, recent studies show that responsive tau phosphorylation not only fails to promote cell apoptosis but also enables resistance to apoptosis. The molecular mechanisms underlying the anti-apoptotic effects of tau hyperphosphorylation are not fully understood but they certainly involve the preservation of β-catenin [283, 284].

β-Catenin is a pro-survival transcription factor. Phosphorylated β-catenin is degraded in the cytoplasm by proteasome-associated proteolysis, while the non-phosphorylated β-catenin is translocated into the nucleus to promote the expression of survival factors. Tau proteins contain 85 potential phosphorylation sites that may compete with β-catenin for the phosphorylation by protein kinases, such as glycogen synthase kinase-3β (GSK-3β). Therefore, the phosphorylation of β-catenin is inhibited with an increased intracellular tau accumulation, leading to the nuclear translocation of β-catenin and cell resistance to apotosis [283].

Lee’s group found that tau protein possesses acetyltransferase activity that can catalyze self-acetylation [285]. We also observed that tau can directly acetylate β-catenin at lysine-49 and inhibit its ubiquitination and phosphorylation, thereby suppressing the cytosolic degradation of β-catenin. The nuclear translocation of β-catenin increases expression of survival factors Bcl2 and survivin, which ultimately enables cell resistance to apoptosis [284]. These findings partially explain why the neurons in AD brains do not undergo massive acute apoptosis even when they are constantly exposed to an increasingly pro-apoptotic microenvironment during AD progression.

Tau hyperphosphorylation enables cells to escape acute apoptosis. Then the cells may restore normal function if the pTau is promptly cleared. However, continuous tau hyperphosphorylation will lead to its intracellular accumulation, which will trigger a series of dysfunctions and eventually lead to chronic neurodegeneration as seen in AD brains. Therefore, the intracellular aggregation of pTau may be a critical step in the transition of tau from anti-apoptotic to pro-neurodegenerative.

#### Aggregation of pTau induces neurodegeneration

The intracellular accumulation of pTau induces neurodegeneration. The mechanisms involve disruption of microtubule assembly and axonal transport, damage to neuronal synapses and neural circuits, induction of subcellular organelle dysfunction, inhibition of proteolysis, and so on.

(1) Aggregation of pTau disrupts microtubules and axons

The recognized function of tau is to promote microtubule assembly and maintain the stability of microtubules, by which it establishes the track for axonal transport [286]. Studies have shown that the level of soluble tau is decreased while the insoluble aggregated tau is significantly increased in the brains of AD patients [287]. The hyperphosphorylated tau not only loses its biological activity in promoting microtubule assembly and maintaining the stability of the microtubules, but also serves as a “seed” to recruit normal soluble tau to form aggregates or take tau protein away from already formed microtubules [288–291]. The pTau can also bind the high-molecular-weight microtubule-associated protein-1 (MAP-1) and MAP-2, and take MAPs away from already formed microtubules, causing microtubule disassembly and eventually collapse [273, 288, 292, 293]. The microtubule collapse induced by pTau aggregation will disrupt axonal track formation and thus damage axonal transport [288, 294–298]. In addition, tau can bind microfilament (such as actin), tyrosine kinase (such as Fyn and Src) [299] in the synapses, histone deacetylase-6 [300], APOE, and other molecules [301–305], by which it affects downstream signaling pathways and cell functions.

(2) Aggregation of pTau impairs synapses and neural circuits

The normal tau is mainly distributed in neuronal axons, while pTau accumulates in the cell body and dendrites, leading to impairment of synapses. For instance, tau aggregation mediates the distribution of tyrosine kinase Fyn in the postsynaptic region, leading to phosphorylation and inactivation of NMDARs [304]. Tau accumulation upregulates Janus kinase 2/signal transducer and activator of transcription 1 (JAK2/STAT1) signaling, and STAT1 can directly bind to the specific GAS element of GluN1, GluN2A, and GluN2B promoters or interact with STAT3 to suppress expression of NMDARs [306, 307]. Tau accumulation disrupts intracellular calcium signaling, leading to activation of calcineurin and dephosphorylation of nuclear CREB, consequently inhibiting glutamatergic transmission and LTP [308], a fundamental feature of learning and memory.

With the development of various neural circuit tracing and manipulating techniques, the circuit mechanisms underlying AD are being revealed [309]. It has been revealed that pTau accumulation in different brain regions and different types of neuron affects neural circuit by different mechanisms. For instance, accumulation of tau within GABAergic interneurons in hippocampal dentate gyrus has a detrimental effect on adult hippocampal neurogenesis. This impairment is caused by the suppression of GABAergic transmission and the subsequent disinhibition of neural circuits within the neurogenic niche [310]. Furthermore, the pathological accumulation of tau in mossy cells induces spatial memory deficits similar to those observed in AD, and this effect is attributed to the inhibition of local neural network activity [311]. Moreover, tau accumulation within the medial septum (MS) cholinergic neurons induces a pronounced impairment in the MS-to-hippocampal CA1 circuit. Notably, those cholinergic neurons exhibiting an asymmetric discharge characteristic, particularly within the MS-hippocampal CA1 circuit, display an increased vulnerability to tau accumulation [312]. The significance of this phonomenon and the underlying mechanism deserve further inverstigation.

In the clinic, AD patients often show emotional or psychiatric symptoms in the early stage with a simultaneous spatial memory deficit. By anterograde and retrograde tracing, we have identified a novel neural circuit, the infralimbic medial prefrontal cortex-posterior basolateral amygdale-ventral hippocampal CA1 (iMPC-pBLA-vCA1) circuit that links emotions to spatial memory and is impaired in AD mice [313–315].

(3) Aggregation of pTau damages suborganelle functions

Mitochondria are the powerhouse of the cell, and mitochondrial dysfunction plays an important role in aging and AD [316, 317]. Accompanying increased tau in the cytoplasm, accumulation of elongated mitochondria has been detected around the cell nucleus [318]. Simultaneously, the mitochondrial membrane potential is increased with impaired energy production and inhibition of mitophagy [319–321]. Unlike the role of Aβ, tau aggregation disrupts mitochondrial fission–fusion by promoting fusion [318, 322], which may also explain why tau hyperphosphorylation has an anti-apoptotic effect. Intracellular aggregation of tau also induces ER stress, Golgi fragmentation, DNA double strain breaking, etc. [323–330].

(4) Aggregation of pTau inhibits autophagy

Autophagy deficits, commonly seen during aging and in AD [331, 332], can induce intracellular tau accumulation. Interestingly, studies show that intracellular tau accumulation can in turn induce autophagy deficits [333, 334].

The increased tau could inhibit autophagosome formation (early steps of the autophagy pathway) by increasing the activity of mammalian target of rapamycin kinase complex 1 (mTORC1), evidenced by the increased levels of p-4EBP1 (phosphorylated eukaryotic translation initiation factor 4E-binding protein 1), p-p70S6K1 (phosphorylated 70 kDa ribosomal protein S6 kinase 1), and p-ULK1 (phosphorylated unc-51-like autophagy-activating kinase 1). The mechanisms involve binding of tau to the prion-related domain of T cell intracellular antigen 1, which increases intercellular amino acids, leading to activation of mTORC1 and inhibition of autophagosome formation [333].

Tau accumulation can also suppress autophagosome-lysosome fusion, the downstream step of the autophagy pathway. The intracellular tau aggregation inhibits the expression of IST1, a positive modulator for the formation of the Endosomal Sorting Complex Required for Transport (ESCRT) complex that is required for autophagosome-lysosome fusion. IST1 facilitates association of CHMP2B (charged multivesicular body protein 2B) with CHMP4B/SNF7-2 to form the ESCRT-III complex, while lack of IST1 impedes formation of the complex. Tau accumulation suppresses IST1 transcription through ANP32A-regulated mask of histone acetylation [334]. These findings together suggest that tau accumulation inhibits autophagy by different molecular mechanisms, which reveal a vicious cycle of tau accumulation and autophagy deficit in the chronic course of AD neurodegeneration.

### Mechanisms underlying tau hyperphosphorylation

Tau protein was first reported as a factor for microtubule assembly in 1975, and it was identified as a neuron-specific cytoskeleton protein in 1985. In 1986, Grundke-Iqbal et al. revealed that the abnormal pTau is the major protein component of the PHF/NFTs isolated from the brains of AD patients [3, 335]. Since then, over 60 phosphorylation sites have been identified in AD brain extracts [269, 270]. Phosphorylation of tau is regulated by protein kinases and phosphatases [336]. Thus, the imbalance between kinases and phosphatases is the direct cause of the hyperphosphorylation of tau proteins.

#### Role of protein kinases in AD-like tau hyperphosphorylation

The full-length human tau protein (441 aa) has 85 potential phosphorylating sites, in which 80 are serine/threonine (Ser/Thr) sites, and 5 are tyrosine (Tyr) sites (Fig. 4).

(1) Ser/Thr kinases

Protein kinases exhibit high diversity and possess complex regulatory mechanisms. Based on the substrate sequence characteristics, Ser/Thr kinases can be classified into two major types, i.e., proline-directed protein kinases (PDPK) and non-proline-directed protein kinases (non-PDPK) [337]. PDPK targets the motif with proline [–X–(S/T)–P], while non-PDPK targets the motif without proline [–X–(S/T)–X–] (where X represents any amino acid, S represents serine, T represents threonine, and P represents proline). Among the known AD-associated phosphorylation sites, approximately half are PDPK sites, while the other half are non-PDPK sites (Fig. 4). The identified PDPKs that can phosphorylate tau protein include ERKs, cell division cycle protein kinase-2, Cdk2, Cdk5, and GSK-3β [338]. The non-PDPKs that can phosphorylate tau protein include cyclic-AMP-dependent protein kinase (PKA), protein kinase C (PKC), calcium/calmodulin-dependent protein kinase II, rat cerebellar calcium/calmodulin-dependent protein kinase (Grkinase), PKN [339], tau tubulin kinases (TTBK) 1 and 2 [340, 341], DYRK [342], MARK [343], Chk1 and 2 [344], casein kinase-1 (CK-1), and CK-2 [345–347]. It is worth noting that the individual phosphorylation efficacy of these kinases on tau proteins may be relatively low. However, pre-incubation of tau with non-PDPKs, such as PKA, CK-1 and PKC, significantly enhances the subsequent phosphorylation rate catalyzed by PDPKs (such as GSK-3β), leading to a significant increase in tau phosphorylation level [348]. This suggests that the phosphorylation of tau catalyzed by PDPKs may be subject to positive regulation by non-PDPKs, and vice versa, which adds complexity to the phosphorylation process in vivo.

(2) Tyrosine kinases

The full-length tau protein has 5 tyrosine residues, i.e., Tyr18, Tyr29, Tyr197, Tyr310, and Tyr394. Among them, only Tyr394 phosphorylation is detectable under physiological conditions, while an increased phosphorylation at Tyr18, Tyr197, and Tyr394 has been identified in the brains of AD patients [349, 350]. Early studies demonstrated that tyrosine kinase c-Abl can phosphorylate tau at Tyr394; TTBK1 can phosphorylate tau at Tyr197; non-receptor tyrosine protein kinases such as SFK (Src family kinase) and Syk (spleen tyrosine kinase) can phosphorylate tau at Tyr18; and kinase Fyn can phosphorylate tau at Tyr18. By binding to Fyn, tau can detain Fyn at postsynaptic sites of excitatory neurons, where Fyn can phosphorylate NMDARs and PSD95, and thus enhance the excitotoxicity of NMDARs [304]. Aβ can activate Fyn to cause synaptic toxicities, and these toxic effects disappear when tau is knocked out [303], which supports an indispensable role of tau in mediating Aβ toxicity on synapses. In addition to Fyn, c-Abl is also present in NFTs and co-localizes with tau [351]. In the early stages of tangle formation, c-Abl levels are increased in neurons. Aβ treatment of primary neurons results in increased c-Abl activity, while intraperitoneal injection of c-Abl inhibitor imatinib mesylate rescues cognitive impairments in AD animal models [352]. These data suggest that tyrosine phosphorylation of tau is involved in AD. However, whether and how tyrosine phosphorylation of tau plays a role in AD neurodegeneration needs further validation.

#### Role of protein phosphatases in tau phosphorylation

According to their structure, composition, substrate specificity, and different activators and inhibitors, mammalian protein phosphatases can be roughly classified into five categories: PP1, PP2A, PP2B, PP2C, and PP5, all of which are expressed in the human brain [353]. When using abnormally phosphorylated tau isolated from the AD brain as a substrate, PP1, PP2A, PP2B, and PP5, but not PP2C, can dephosphorylate tau at multiple sites and restore the microtubule assembly activity of tau proteins to varying degrees [354]. Treatment of cultured cells with protein phosphatase inhibitors increases tau phosphorylation with simultaneous alterations in intermediate filament structure, loss of microtubules, and impairments of neuronal synapses and dendrites. The dephosphorylation activity of PP2A and PP2B towards the AD tau can be activated by Mn^2+^ and Mg^2+^, with Mn^2+^ having a stronger effect than Mg^2+^ [355]. The PP2B activity on tau dephosphorylation is also enhanced by Ca^2+^/calmodulin [356]. The dephosphorylation effects of the phosphatases on the insoluble PHF/NFT are weaker than the effects on soluble tau proteins. The details for tau dephosphorylation catalyzed by various phosphatases are as follows.

(1) PP2A

PP2A is a heterotrimer consisting of structural subunit A, regulatory subunit B and catalytic subunit C. The B subunit has four subfamilies and is encoded by 15 genes, including at least 23 isoforms. PP2A-ABαC, concentrated in the cytoplasm with small amounts in mitochondria and microsomes, is the major brain form of PP2A involved in tau dephosphorylation. PP2A binds to different sites in tau and microtubules, and changes in either component will affect tau dephosphorylation by PP2A and thus change the structure and function of microtubules.

The evidence for the involvement of PP2A in the AD-like tau phosphorylation is as follows. Reduced PP2A activity is observed in the brains of AD patients. Compared to PP1, PP2B, and PP5, PP2A is the major tau phosphatase accounting for over 70% of AD-like tau dephosphorylation in the brain tissue [357]. Furthermore, PP2A exhibits the highest specific activity in dephosphorylating tau, and thus disassembles tangles and releases free tau protein to restore biological activity of tau [358]. Inhibition of PP2A leads to AD-like hyperphosphorylation of tau protein and disruption of the cellular cytoskeleton, accompanied by impairments in spatial learning and memory in rats. In the brains of AD patients, endogenous PP2A inhibitors, I1PP2A, I2PP2A and CIP2A [359], are colocalized in the cytoplasm with PP2A within neurons, and increasing these inhibitors suppresses PP2A activity and induces tau hyperphosphorylation.

(2) PP2B

PP2B, also known as calcineurin, is the most abundant calcium-dependent phosphatase accounting for ~ 10% of the total protein in the brain. Although PP2B is highly enriched in the brain. In vitro studies demonstrated that the specific activity of PP2B on tau proteins is much lower than that of PP2A [358]. PP2B is primarily localized in the perinuclear region and dendrites of neurons, existing as a heterodimer consisting of a 61 kDa catalytic subunit (C_A_) that can bind to calmodulin (CaM) and a 17 kDa regulatory subunit (R_B_) that can bind to Ca^2+^. There are two isoforms of PP2B, A_a_ and A_b_, with A_a_ being the predominant form in the brain. PP2B requires binding of C_A_ and R_B_ to exert functions and Ca^2+^-CaM, Mn^2+^ and Ni^2+^ activate the phosphatase. Purified PP2B from human brains can dephosphorylate pTau at multiple AD-associated sites [360], and PP2B knockout induces tau hyperphosphorylation with abnormalities of the cytoskeleton [361]. Reports on PP2B activity in the AD brain are contradictory, with some showing an increased PP2B activity rather than a decreased activity.

(3) PP1

PP1 is a complex composed of a catalytic subunit C and various regulatory subunits. It is widely expressed in the plasma membrane, cytoplasm, and subcellular organelles of pyramidal neurons. PP1 may be involved in learning and memory processes by regulating synaptic transmission and plasticity [362–364]. Tau, as an anchor protein, can interact with both PP1 and microtubule, and thereby modulate the phosphorylation state of tau [365]. In AD patients, PP1 activity is decreased [357, 366]. Although in vitro studies indicate the involvement of PP1 in tau dephosphorylation, little is known regarding the in vivo role of PP1 in dephosphorylating tau proteins. It is worth noting that kinase upregulation by PP1 (also applicable to other PPs) may counterbalance its dephosphorylating effect on tau proteins. The main physiological inhibitors of PP1 include inhibitor-1 (I-1), I-2, and I-3 [367]. Okadaic acid (OA, Ki 100 nM) and calyculin A (CA, Ki 50 nM) can also inhibit PP1 [368].

(4) PP5

PP5, highly expressed in neurons of the brain, is capable of dephosphorylating tau at multiple sites in vitro, and the activity of PP5 is decreased in the brains of AD patients [369, 370]. Currently, the role of PP5 in tau dephosphorylation in vivo or its involvement in AD is unclear.

#### Other factors that indirectly influence tau phosphorylation

In addition to protein kinases and phosphatases which directly phosphorylate or dephosphorylate tau proteins, other factors, such as PS1, APOE, etc., have been reported to influence tau phosphorylation indirectly. For instance, PS1 can directly bind to GSK-3β, one of the most prominent kinase in phosphorylating tau at multiple AD-associated sites, and thereby decrease the kinase activity [371]. PS1 can form a complex with β-catenin, by which it enhances the stability of β-catenin and affects tau phosphorylation through Notch and Wnt signaling. In those AD patients with *PSEN1* mutations, both the stability and the level of β-catenin are significantly reduced [372]. Since both β-catenin and tau are substrates of GSK-3β, decreased β-catenin may contribute to an increased tau phosphorylation by substrate competition [283]. *PSEN1* mutations can also change the intracellular transport of β-catenin, thereby affecting tau phosphorylation [373]. In addition, many studies demonstrate that the *APOE*4 genotype is associated with tau hyperphosphorylation compared with *APOE*2 and *APOE*3, although conflict results have been reported.

### Mechanisms underlying tau aggregation

The molecular mechanisms underlying the abnormal aggregation of tau remain unclear. Studies have demonstrated that characteristic motifs, such as hexapeptides, or site-specific phosphorylation in tau proteins could promote tau aggregation. In addition, pTau proteins undergo multiple additional modifications such as glycosylation, glycation, acetylation, nitration, SUMOylation, truncation, methylation, and ubiquitination, and these post-translational modifications can also promote tau aggregation.

#### The aggregation propensity motif in the primary sequence of tau

Although tau itself has no specific spatial structure, the N- and C-terminal in the primary sequence of tau can fold over in the proximity to the center of the microtubule-binding domains to form a “paperclip-like” conformation. In full-length 4R tau, the two hexapeptides, 275VQIINK280 [374] and 306VQIVYK311 [375] (also known as PHF6) located at the initial sequence of 2R and 3R domains, respectively, can drive β-sheet formation. Tau phosphorylation proximal to these regions is relevant to tau aggregation. By using this motif-characteristics, K18 that is composed of the 4R domain of tau or the K18 mutant containing K280 deletion or P301L mutation (a point mutation of tau found in FTDP patients) has been used as a “seed” to efficiently promote tau aggregation [376–378].

Studies also show that the site-specific phosphorylation can directly affect the conformation of tau, thus contributing to the propensity of tau to aggregate [379–381]. For instance, phosphorylation at S202/T205/S208 within the proline-rich region (PRR) induces tau self-aggregation [382]. It is also reported that phosphorylation of tau at S198, S199, S416, S396, and S422 correlates with increased oligomerization or increased aggregation of tau proteins [383]. These data together suggest that the site-specific phosphorylation of tau may play a role in its self-aggregation, and the mechanisms involve changes in charges and conformations of tau proteins. However, it is still not clear which phosphorylation site(s) is indispensable for tau aggregation. As K18 or the mutant could efficiently mimic the AD-like tau aggregation and the cytotoxicity, the K18 models may be used for in-depth mechanistic studies and tau-targeted drug screening.

#### Interplay of different post-translational modifications

(1) Mutual promotion of phosphorylation and SUMOylation aggravates tau aggregation

Small ubiquitin-like modifier (SUMO) is a small ubiquitin-like protein that can reversibly modify substrate proteins in a manner similar to ubiquitination, a process known as SUMOylation [384]. This modification regulates the biological activity, subcellular localization, and stability of proteins. SUMO-1 has been found to co-localize with aggregated phosphorylated tau in the brains of AD patients and mouse models, and the level of SUMO-1 is also increased in the plasma of AD patients [385–387].In vitro studies demonstrate that tau protein at Lys340 can undergo SUMOylation, and SUMOylation of tau at Lys340 reciprocally promotes its phosphorylation at multiple AD-associated sites. As SUMOylation and ubiquitination generally occur at the same alkaline amino acid, SUMOylation of tau inhibits its ubiquitination, thus blocking its degradation by the proteasome system and leading to tau aggregation. Treatment of primary neurons with Aβ results in increased SUMOylation and phosphorylation of tau proteins [388].

(2) Aberrant acetylation and ubiquitination induce tau aggregation

In purified soluble phosphorylated tau from the AD brain, at least 19 acetylation sites have been detected, some of which overlap with the ubiquitination sites [270]. Meanwhile, more and more acetylation sites in tau are being reveled by in vitro experiments [389, 390] (Fig. 4). Ubiquitination occurs more frequently in the microtubule-binding repeats R1–R3 regions, while many acetylation sites are located in the R4 region [270, 389]. Furthermore, mass spectrometry analysis of AD brain samples revealed that both ubiquitination and acetylation occur in the late stages of tau pathology (Braak stages V–VI) and are directly associated with the seeding and aggregation properties of tau proteins [390, 391]. However, studies have also shown that acetylation of certain sites, such as Lys174, occurs in the early-to-mid stages of AD pathology [392]. In cellular experiments, histone acetyltransferase p300 can induce tau acetylation, while SIRT1 (a deacetylase) can deacetylate tau proteins [390, 393].

Interestingly, tau itself possesses acetyltransferase activity that can catalyze its self-acetylation [285]. Tau can also acetylate other substrates, such as β-catenin and GSK-3β [284, 394]. Due to the occurrence of acetylation and ubiquitination at lysine residues, increased acetylation of tau protein can lead to decreased ubiquitination level, resulting in reduced degradation. Simultaneously, acetylation interferes with the binding of tau to microtubules and promotes tau aggregation [390]. Tau acetylation and ubiquitination are both increased in the brains of AD patients, suggesting that abnormal acetylation may precede abnormal ubiquitination. Acetylation competes for the lysine residues, preventing effective ubiquitination and degradation of tau, leading to tau aggregation [395].

Ubiquitin, a peptide consisting of 76 amino acids, is covalently attached to target proteins via an isopeptide bond between its C-terminal glycine and a lysine residue of the substrate [396]. Under normal conditions, ubiquitinated proteins are degraded through the ubiquitin–proteasome pathway, where they are recognized and proteolised by proteasome. However, deregulation of the ubiquitin degradation pathway or structural changes of the targeted protein will make them less susceptible to ubiquitination, leading to impeded degradation and increased protein aggregation [397]. Interestingly, ubiquitin is significantly increased in AD brains and is primarily detected in the insoluble PHF/tangle aggregates [398]. To date, at least 28 potential ubiquitination sites on tau have been identified [399], in which 17 are exclusively present in the insoluble tau aggregates, and 16 are located in the microtubule-binding region [270, 400] (Fig. 4). The elevated ubiquitination of tau protein in the AD brains may be a consequence of its aggregation, or represent a compensatory response to degrade the abnormally aggregated tau proteins. Although the relationship of ubiquitination with acetylation, SUMOylation, and phosphorylation is still not clearly elucidated, a competition between ubiquitination and acetylation/SUMOylation has been recognized [388, 401].

During the process of tau aggregation, the microtubule-binding domain (MBD) forms the core of the aggregates through β-folding. The MBD contains 19 lysine residues which, under physiological conditions, carry a positive charge that prevents β-folding due to electrostatic repulsion. Phosphorylation of serine, threonine, and tyrosine residues in the MBD, as well as acetylation of lysine residues, may promote β-folding through a charge neutralization effect by introducing negative charges. Additionally, phosphorylation is also frequently observed in the PRR, which is believed to facilitate aggregation by spatially approaching the MBD and neutralizing its positive charges [270].

In addition, methylation of tau at arginine residues, primarily occurring in the MBD, has been revealed from both healthy and AD brains [267, 402–405] (Fig. 4). Some lysine residues (K163, K174, and K180) can be both acetylated and methylated in vivo. The exact impact of lysine methylation on endogenous tau activity remains unclear [404]. The in vitro methylated tau appears to have reduced aggregation tendencies and can promote tubulin assembly, hinting at a protective role against protein aggregation [405]. In the context of AD, methylated residues on tau aggregates raise the intriguing possibility of interfering with ubiquitination and impeding proteasome-mediated degradation [402], which may deserve further examination.

#### Glycosylation and glycation may differently affect tau aggregation

Glycosylation refers to the process in which specific glycosyltransferases covalently attach sugar moieties to protein molecules, forming glycoproteins through N-glycosidic or O-glycosidic bonds. In normal tissues, protein glycosylation occurs in the rough ER and Golgi apparatus during protein synthesis, either during translation (N-glycosidic bonds) or post-translationally (N- and O-glycosidic bonds) [406, 407]. The glycosyltransferases involved in this process are often membrane-bound. Tau is a cytosolic protein. Glycosylation of tau often indicates abnormalities in the membrane structure, which allows for interaction between tau and glycosyltransferases. The presence of abnormalities of membrane lipids and membrane fluidity in AD patients supports this hypothesis. Furthermore, the AD-related PS1, PS2, and APP are all membrane-associated proteins, and PS1 and PS2 are highly expressed in rough ER and Golgi apparatus. Therefore, exploring the relationship between abnormal glycosylation of tau and abnormalities of membrane proteins (PS and APP) is of significance for elucidating the mechanisms underlying AD pathogenesis.

In AD, the brain-derived PHF/tangles and the pTau are subjected to abnormal glycosylation [408, 409]. These modifications predominantly involve the addition of terminal mannose, sialic acid α-(2–3)-linked to galactose, β-galactose (1–3)-N-acetylglucosamine, and β-galactose (1–4)-N-acetylglucosamine, with N-glycosylation being the primary form. When PHF/NFT is subjected to negative staining electron microscopy in the presence of glycosidases at 37 °C, the observed PHF structures disappear, and instead, more compact and elongated fiber-like structures are formed [408]. However, deglycosylation alone does not restore the biological activity of tau, nor does it significantly increase the release of tau protein from PHF/NFT. Nevertheless, subsequent treatment with PP2A to dephosphorylate the deglycosylated tau significantly increases tau release from tangles compared to dephosphorylation alone. This suggests that the abnormal aggregation of tau in the AD brain can be reversed. Hyperphosphorylation of tau primarily contributes to the formation and stability of PHF/NFT, while tau glycosylation may contribute to the maintenance of the helical filament structure [410].

There are conflicting reports regarding the relationship between O-glycosylation and phosphorylation of tau protein [411, 412]. The pTau is O-glycosylated in the AD brain samples, but in vitro studies show coexistence of low levels of O-glycosylation and phosphorylation in tau [413, 414]. Other studies showed that fasting induces a time-dependent reduction of protein O-glycosylation with a simultaneous increase in protein phosphorylation, suggesting that the impaired glucose metabolism in the AD brains may induce tau hyperphosphorylation by decreasing O-glycosylation [413, 415]. On the other hand, self-aggregation of pTau may create a “seeding site” within affected neurons, and the increased intracellular concentration of phosphate group caused by hyperphosphorylation may make tau more susceptible to glycosylation. Therefore, it can be inferred that hyperphosphorylation may promote tau glycosylation.

In addition, tau protein in the AD brains undergoes abnormal glycation [416, 417]. Glycation refers to the process in which the ε-NH2 group of protein molecules reacts with the aldehyde groups of intracellular sugars, forming Schiff bases through oxidation without requirement of enzymes. Subsequent intramolecular rearrangement leads to the formation of insoluble, protease-resistant, and irreversible cross-linked structures known as advanced glycation end products (AGEs) [418]. Tau proteins, enriched in lysine and ε-NH2, are highly susceptible to AGE formation [419, 420]. The formation of AGEs may contribute to the transition of PHFs to NFTs, leading to irreversible damage to neurons [417, 420].

#### Site-specific tyrosine nitration differently affects tau aggregation

Nitration of tau protein has been observed in NFTs and tau inclusions in AD patients (Fig. 4), suggesting the involvement of tau nitration in its aggregation [421]. Treatment of tau with peroxynitrite (ONOO-) in vitro leads to 3-nitrotyrosine (3-NT) immunoreactivity and the formation of SDS- and heat-stable oligomers through dityrosine cross-linking. This 3-NT-modified tau is elevated in the brains and CSF of AD patients [422, 423]. Tau contains five tyrosine residues, Tyr18, Tyr29, Tyr197, Tyr310, and Tyr394 [424]. Nitration of tau at Tyr197 has been detected in both normal individuals and AD patients. Nitration at Tyr29 has been detected in both soluble and insoluble tau proteins from AD patients but not in normal brain tissue. Nitration at Tyr394 has been detected only in the insoluble PHF-tau derived from AD patients [425, 426]. In addition, the nitration of tau at Tyr18 is predominantly observed in the activated astrocytes in AD brains [427]. Treatment of tau with peroxynitrite in vitro results in nitration primarily at Tyr18 and Tyr29, and nitration at these sites inhibits tau aggregation [428]. With these conflicting data, the specific role and mechanism of nitration at different sites during AD progression, as well as the relationship between tyrosine phosphorylation and nitration at the Tyr sites, are currently unclear.

#### Truncation at various sites differently affects tau aggregation

Tau truncation refers to the enzymatic cleavage of tau proteins at its N-terminus or C-terminus, resulting in production of shorter molecular forms of tau. At least two classes of enzymes have been reported to cleave tau: the caspase family members and AEP [429].

(1) Tau truncation by caspases

Caspases are key enzymes in the apoptosis pathway. The activities of caspases-2 and -3 are increased in AD brains [430], but their roles in the induction of neuronal apoptosis in AD are still unclear [17]. Both in vitro and in vivo experiments have shown that caspases-2, -3, and -6 can cleave tau protein at Asp421, Asp418, Asp314, and Asp13 [431, 432]. The truncated forms of tau protein generated by cleavage exert toxic effects on neurons through different mechanisms [433–435]. For example, tau truncated at Asp421 by caspases-3 and -6 damages microtubules and cytoskeleton [434, 436]. Tau truncated at Asp314 by caspase-2 results in mislocation of tau into the dendritic spines and impairs the function of postsynaptic membrane glutamate receptors [432, 437], although it is also reported that the Asp314 truncation exhibits anti-aggregation properties [432, 437].

(2) Tau truncation by AEP

AEP, an endopeptidase that can cut peptide bond formed by asparagine, is primarily found in lysosomes and it is activated under acidic conditions [438, 439]. The activity of AEP is increased in the brains of aged and AD patients. An in vitro study demonstrated that AEP could cleave tau protein at Asn255 and Asn368, generating truncated forms of tau (1–368 and 256–368); and both truncated forms of tau exhibit reduced microtubule assembly capacity and are toxic to neurons [213]. Truncation of tau (especially at C-terminal) induces conformational changes of tau, by which it results in proximity of the proline-rich region to the microtubule-binding domain and thus promotes tau aggregation [440].

#### Sequential order of different post-translational modifications of tau

The role of different post-translational modifications in tau aggregation has been constantly revealed, but their sequential order is still unclear. A recent study analyzed the occurrence and frequency of different post-translational modifications at various tau sites in brain tissues from AD patients at different Braak stages and from control subjects [270]. Results showed that partial phosphorylation of tau protein occurs in the PRR and C-terminal at Braak stages 0–2; more phosphorylation sites are detected in these two regions at Braak stages 3–4; acetylation and ubiquitination occur in the MBD in addition to the increased phosphorylation sites in Braak stages 5–6; and various post-translational modifications significantly increase in the most severe AD patients [383]. It is speculated that phosphorylation of tau in the PRR and C-terminal region may initially change its charge and conformation, which promotes β-sheet formation in the MBD and serves as the core for tau aggregation [375]. Tau aggregation may increase its lysine acetylation which could aggravate tau accumulation by neutralizing the positive charge in the MBD region. Tau accumulation can activate the ubiquitination system to degrade tau proteins, which may explain why increased ubiquitination has been often detected in the NFTs of AD brains. In addition, truncation of tau also enhances its aggregability, which may play a role in initiating aggregation and promoting the pathological spreading of tau proteins in the early stages [441].

#### Tau gene mutations promote its phosphorylation and aggregation

To date, no gene mutation on tau has been found in AD patients. However, over 50 distinct mutations in tau gene have been identified in individuals with FTDP-17 [442, 443]. These mutations encompass various types, such as missense mutations, deletion mutations, silent mutations, and intronic mutations downstream of exon 10 within the coding regions of exons 9, 10, 12, and 13. Functionally, these mutations exert their effects at both RNA and protein levels [444], resulting in diminished microtubule-binding affinity of tau protein, enhanced aggregation propensity [445, 446], and an altered ratio of 4R-tau to 3R-tau isoforms, ultimately culminating in elevated levels of 4R-tau species within the brain [447]. Compared to the wild-type tau, the mutated tau proteins associated with FTDP-17 exhibit increased susceptibility to hyperphosphorylation and aggregation, thereby displaying an augmented cytotoxicity [448].

Tau mutations in FTDP-17 may contribute to increased phosphorylation through the following mechanisms. First, the mutations alter the conformation of tau to a more favorable substrate for protein kinases. Consequently, tau proteins with mutations are more prone to hyperphosphorylation. Second, several mutations in tau reduce its binding affinity with PP2A, a crucial phosphatase in tau dephosphorylation [449]. Furthermore, the mutations facilitate tau self-aggregation even at lower levels of phosphorylation [446].

There are still many other factors affecting tau phosphorylation and aggregation. For instance, heat shock protein 90, one of the major tau-binding chaperones, promotes tau phosphorylation and aggregation by binding tau at the VQIVYK motif and inducing conformational changes on tau [450]. On the other hand, Hsp70 and Hsp104 show inhibitory effects on tau seeding and aggregation [451, 452].

### Mechanisms underlying tau transmission

In AD patients, tau pathology as NFTs first appears in the entorhinal cortex, and then spreads from the hippocampal limbic system to the whole brain. Based on this sequential appearance, brain pathology of AD patients can be classified into six Braak stages [453]. Currently, it is not clearly understood how the abnormal tau proteins propagate into different brain regions or are transmitted from one cell to another. Studies suggest that the transcellular tau propagation may follow the prion-like transmission pattern [454–456], but the exact mechanism underlying prion transmittion is also not clear. As tau is an intracellular protein, the transmission process should at least involve the release of intracellular tau to the interstitial space and the uptake of extracellular tau by recipient cells.

#### Release of intracellular tau proteins into the interstitial space

As a cytoskeleton protein, tau is distributed in the intracellular compartment, mainly in neuronal axons in physiological conditions [457, 458]. In vitro studies demonstrate that tau can be actively released into the extracellular spaces through synapses or extra-synaptic compartments, which explains the detection of tau proteins in the extracellular matrix by microdialysis or in the CSF, though the level is relatively low in physiological conditions. The CSF level of tau, especially the pTau proteins, is significantly increased in a majority of AD patients, suggesting increased tau release in AD [459–461]. These results indicate that tau proteins may be secreted by presynaptic neurons, and the secretion is significantly increased in AD patients, leading to increased tau transmission.

The trans-synaptic tau spreading is achieved by direct transmission of exosomes between neurons in both physiological and pathological conditions [462]. Interestingly, the neuron-derived exosomal tau is less phosphorylated than the tau retained in the cytosol. Whether this type of secretion has any significance is unclear [463]. It is also found that neuron depolarization or synaptic activity promotes the release of tau-containing exosomes, which highlights the importance of neuronal activity in tau transmission [462].

In addition, the increased interstitial level of tau during AD progression may also be attributed to the impaired integrity of the neuronal plasma membrane, which leads to a massive leak of tau into the extracellular space. This speculation may deserve further investigations.

#### Uptake of extracellular tau by the recipient cells

Cells in the central nervous system can also actively internalize monomeric tau via receptor-mediated endocytosis and non-receptor-mediated pathways. For the receptor-mediated endocytosis, it has been reported that the monomeric tau is internalized by neurons via muscarinic receptors M1 and M3 [464], while the uptake of tau by microglia is mediated by CX3CR1 [465, 466]. In astrocytes, uptake of tau-containing exosomes has not been detected [462]; instead, non-HSPG-dependent endocytosis of tau proteins has been shown [467]. The non-receptor-mediated pathways include HSPG-associated macropinocytosis [468], clathrin-mediated endocytosis and bulk endocytosis [469]. LRP1, which is abundantly expressed in neurons, microglia and astrocytes, has also been identified as a major regulator of tau spreading in the brain [470].

**Fig. 5:**
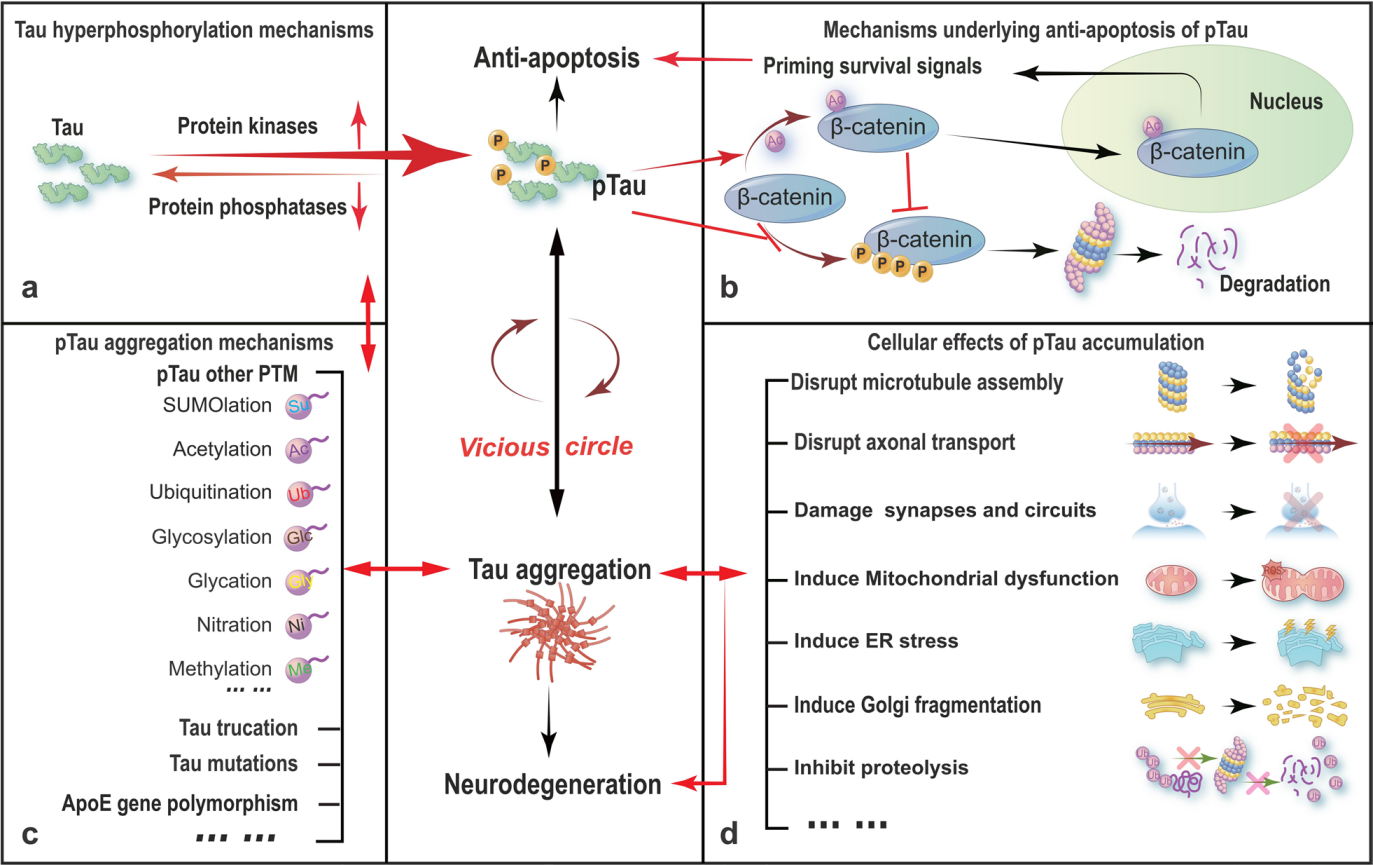
Mechanisms underlying tau-associated neurodegeneration in AD. **a** Imbalance of protein kinases and protein phosphatases leads to tau hyperphosphorylation. **b** Tau hyperphosphorylation inhibits acute cell apoptosis by preserving β-catenin. **c** Additional post-translational modifications of pTau promote its intracellular aggregation and accumulation. **d** Abnormally accumulated tau protein impairs multiple cellular biological functions. Tau hyperphosphorylation and accumulation forms a vicious cycle, resulting in chronic neurodegeneration as seen in AD

In summary, during the course of AD, dysregulation of protein kinases and phosphatases leads to tau hyperphosphorylation; increased tau phosphorylation inhibits the phosphorylation of β-catenin with mechanisms involving direct acetylation of β-catenin by tau, or through substrate competition of tau and β-catenin for the kinases (such as GSK-3β). The increased acetylation inhibits ubiquitination of β-catenin, which together with the reduced phosphorylation, makes β-catenin escape from proteolysis and retain in the cytoplasm. The increased cytoplasmic β-catenin is translocated into the nucleus with currently unknown mechanism and promotes expression of survival factors, such as Bcl2 and survivin. Finally, the cells escape from an acute apoptosis. If pTau can be timely dephosphorylated, the tau-related neurodegeneration can be prevented [29]. Most importantly, as dephosphorylation of tau proteins can restore their biological functions in promoting microtubule assembly and stability, it may improve the architecture of cytoskeleton and thus promote outgrowth or regeneration of the dystrophic neuronal processes as seen in AD, thereby curing AD neurodegeneration. If the pTau proteins are not timely handled, they may undergo various other post-translational modifications such as acetylation, SUMOylation, glycosylation, glycation, nitration, and truncation, etc. One of the adverse consequences of these modifications is the intracellular aggregation of tau proteins. Tau aggregation can disrupt microtubule assembly and axonal transport, induce dysfunctions of mitochondria, ER, Golgi, synapses and neural circuits, and inhibit proteolysis. The vicious cycle between tau hyperphosphorylation and aggregation continuously aggravates tau accumulation, propagation, and neural damage, ultimately leading to chronic neurodegeneration (Fig. 5).

## AD experimental models and their characteristics

Animal models that replicate human diseases are invaluable for investigating the underlying mechanisms and developing new drugs [471]. Currently, over a hundred AD animal models have been reported, including mouse, rat, and non-human primate models. Among them, the mouse models are the most widely used. The basic features of AD mouse models include varying degrees of cognitive impairments at different ages, accompanied by synaptic damage, Aβ elevation or plaque formation, tau hyperphosphorylation and aggregation, and activation or proliferation of glial cells. Although the use of animal models has made significant contributions to unraveling the disease mechanisms, their effectiveness in drug development has been somewhat disappointing. In recent years, scientists have attempted to establish AD patient-derived cell and organoid models that better simulate their brain pathology [472].

### AD mouse models

#### Aβ-related models

Current AD mouse models mainly focus on the *APP* and *PSEN1* gene mutations detected in AD patients [473–476]. Based on the types of mutated genes expressed in AD mice, the Aβ-related mouse models can be broadly categorized as follows.

(1) Models expressing various *APP* mutations

These include PDAPP (AβPPInd) [477], Tg2576 (AβPPSwe) [478], APP23 (AβPPSwe) [479], TASD-41 (AβPPSwe, Lon) [480], J20 (AβPPSwe, Ind) [481], TgCRND8 (AβPPSwe, Ind) [482], and others [483]. Heterozygous PDAPP mice develop sulfur-positive Aβ deposits and neuroinflammatory plaques between 6 and 9 months of age [477]. Among them, the Swedish mutation (Swe, K670N/M671L) located near the N-terminus of Aβ and promoting β-cleavage, is the most common. The Indiana (Ind, V717F), London (Lon, V717I), Florida (Flo, I716V), and Iberian (Ibe, I716F) mutations are located near the C-terminal γ-cleavage site of Aβ and promote γ-cleavage of APP, resulting in the production of longer, more toxic Aβ molecules. The Dutch mutation (Dutch, Aβ E22Q), Arctic mutation (Arc, Aβ E22G), and Iowa mutation (Aβ D23N) are internal mutations within the Aβ peptide, altering the amino acid sequence of Aβ [484–487].

(2) Models simultaneously expressing mutated *APP* and *PSEN1* genes

The PS/APP (AβPPSwe/PS1M146L) [488, 489], 2KI (AβPPSwe/PS1P264L) [490] and 5 × FAD (AβPPSwe, Lnd, Flo/PS1M146L, L286V) [491, 492] are the most commonly used models [474, 493]. The APP/PS1 mice exhibit cortical amyloid plaque deposition and glial activation at 4 months of age, along with reduced synaptic numbers in the hippocampus [490]. They also experience significant spatial learning and memory impairments at 6 months of age. In these mice, the extent of amyloid pathology is higher in females than in males. 5 × FAD mice show intraneuronal Aβ aggregation at 1.5 months of age, followed by amyloid deposition and glial activation at 2 months, and subsequently synaptic, neuron loss, and cognitive impairment at 4–5 months of age [493].

(3) Models simultaneously expressing mutated *APP*, *PSEN1*, and *MAPT* genes

The 3 × Tg AD (AβPPSwe/tauP301L/PS1M146V) [494–496] mice exhibit brain amyloid deposition at 3–4 months, impaired synaptic transmission and LTP at 6 months, and hippocampal tau hyperphosphorylation at 12–15 months of age; it is also reported that 3 × Tg AD mice show tau hyperphosphorylation, neuroinflammation, and reduced cognitive function at 6 months of age. The APP/PS1 and 3 × Tg AD mouse models exhibit varying degrees of tau pathology at certain age stages, but they do not form NFTs [476]. In addition, AβPPSwe and Ind mice co-transfected with APOE4 [497] or co-regulated with leptin knockout (ob/ob) are also useful models for AD research [483].

We can also replicate AD-like pathologies and behavioral changes by intraventricular or bilateral hippocampal injection of Aβ oligomers or fibers [498]. Additionally, brain region- or neural cell type-specific gene manipulation to mimic AD-like pathologies can be achieved through viral vector transduction or crossing with specific Cre mice.

#### Tau-related models

To date, no tau gene mutations have been found in AD patients, and tau pathology in AD is primarily caused by intracellular accumulation of wild-type tau proteins with different types of post-translational modifications.

(1) Wild-type tau models

The transgenic mice expressing human full-length wild-type tau with knockout of endogenous murine tau (PAC transgenic × MAPT KO, JAX005491) [499–502] show aggregation of insoluble pTau at 9 months of age, with spatial- and temporal-dependent progressive synaptic damage, behavioral impairment, and neuronal loss. However, the pathological and behavioral characteristics in this line gradually weaken during continuous passaging, and the mice show significantly decreased reproducibility, possibly due to copy number dropping of the gene. Mice expressing the human wild-type 0N3R tau under the control of the mouse *Prnp* promoter (JAX 003741) also show age-dependent pathological and behavioral changes [503].

(2) Mutant tau models

Mice expressing wild-type tau need a long time to exhibit AD-like changes, while tau mutants identified in FTDP-17 (such as P301L and P301S) induce AD-like neuropathology and cognitive deficit in a shorter time. Therefore, tau-P301L and tau-P301S are more widely used tau models. As P301L-tau0N4R (Tg4510) is unable to produce overt brain atrophy, a bi-transgenic mouse model rTg4510 (“r” refers to regulatable) with CaMK2α promoter was established by crossing Tg4510 to a driver line that harbors a tetracycline transactivator transgene [504, 505]. The homozygous progeny of the rTg4510 express human P301L-0N4R-tau specifically in the forebrain neurons, and show spatial memory deficits at 3–5 months and neuronal loss at 8 months of age [505]. These mice also show serious motor impairments, which may disturb the evaluation of cognitive functions in behavioral tests [506].

In addition, mice expressing human P301S-1N4R-tau driven by the PrP promoter on a B6/C3 background (PS19, JAX008169) show hippocampal synaptic loss and impaired synaptic function at 3 months. The filamentous tau lesions appear at 6-month, and progressive tau accumulation associated with neuron loss and hippocampal and entorhinal cortical atrophy are detected by 9–12 months of age. The mice show spatial learning and memory impairments at 7 months of age, and NFT formation and gliosis can be observed at 8 months of age [507].

(3) Truncated tau model

To better mimic the AD-like tau pathologies (i.e., wild-type tau, accumulating at adult but not the embryonic stage), we recently developed a new tet-on transgenic mouse model expressing truncated human tau N1-368 (termed hTau368), a tau fragment increased in the brains of AD patients and the aged mice. Dox treatment of the hTau368 transgenic mice at a young age for 1–2 months is sufficient for inducing overt and reversible human tau accumulation in the brain, predominantly in the hippocampus, together with the AD-like high level of pTau, glial activation, neuronal loss, impairment of hippocampal neurogenesis, synaptic degeneration and cognitive deficits [508].

Non-transgenic approaches have also been used to produce brain tau pathology, such as brain stereotaxic infusion of viral vectors containing different tau genes (commonly AAV1, 2, 6, 9, or lentivirus) into the brains of animals. These vectors can express different isoforms of tau or mixtures thereof, tau with specific phosphorylation site mutations, various tau truncations, etc. By embryonic brain injection of AAV1 viral vectors, global expression of different tau genes can be achieved. Additionally, high-aggregation PHF-tau protein extracted from AD patient brains can be injected into the brains of wild-type or tau/APP transgenic animals to simulate AD pathologies. All of these modeling methods can induce AD-like pathological and/or behavioral changes at different ages or time points after expression.

Although tau knockout mice may exhibit pathological changes such as intraneuronal iron accumulation in the later stages of aging, it seems not significantly impact mouse development and survival, suggesting the presence of compensatory mechanisms for the function of tau proteins.

Table 1 provides a summary of commonly used AD transgenic mouse models and their characteristics. These models are being extensively used in investigating AD mechanisms and developing drugs targeting pathological Aβ and tau, as well as their associated pathological and behavioral changes (http://www.alzforum.org/research-models).

**Table 1:** Several commonly used transgenic mouse models of AD

Model Name	Transgene (Promoter)	Isoform	Genetic background	Characteristics	References
PDAPP	hAPP_Ind( PDGFβ)_	hAPP_695,751,770_	C57BL/6 × DBA2	Cognitive impairment, deposition of ThS-positive Aβ, neuroinflammatory plaques, synaptic loss, and proliferation of astrocytes and microglia after 3 months	[477]
Tg2576	hAPP_Swe( PrP)_	hAPP_695_	C57BL/6 × SJL	After 6 months, the mice have impaired learning and memory. Abundant Aβ plaques are observed by 11–13 months	[478]
APP23	hAPP_Swe( Thy1)_	hAPP_751_	C57BL/6 J	After 3 months, age-related spatial memory deficits become pronounced. By the sixth month, amyloid plaques and elevated p-tau levels manifest, all in the absence of neurofibrillary tangles	[479]
TASD-41	hAPP_Swe/Lon( Thy1)_	hAPP_751_	C57BL/6 J x DBA	Plaques appear in the frontal cortex at 3–6 months and spread with age, leading to cognitive impairment by 6 months	[480]
J20	hAPP_Swe/Ind(PDGFβ)_	hAPP_695,751,770_	C57BL/6 × DBA/2	Diffuse Aβ plaques begin at 5–7 months, becoming widespread by 8–10 months. Mice show spatial reference memory deficits by 4 months old	[481]
TgCRND8	hAPP_Swe,Ind(PrP)_	hAPP_695_	Hybrid C3H/He- C57BL/6	At 3 months, cognitive deficits emerge in Morris water maze learning. Amyloid deposition begins, leading to plaques by 3 months. Between 7–12 months, tau undergoes hyperphosphorylation, but neurofibrillary tangles remain absent	[482]
PS/APP	hAPP_Swe(PrP)_;hPS1_M146L(PDGFβ)_	hAPP_695_/hPSEN1	B6/D2/Swe/SJL	Aβ accumulates in the cortex and hippocampus starting at 6 months, increasing with age. Hyperphosphorylated tau appears at 24 weeks, with no neurofibrillary tangles. Cognitive deficits emerge at around three months and worsen with age	[488, 489]
2xKI	hAPP_Swe(PrP)_;mPS1_P264L(PS1)_	hAPP_695_/m PSEN1	129 × Tg2576	At one month, mice exhibit elevated Aβ42/Aβ40 ratios. Bigenic mice also display accelerated amyloid deposition (around four months) and heightened reactive gliosis compared to Tg2576 mice	[490]
5xFAD	APP_Swe/Lond/Flo(Thy1)_;PS1_M146L,L286V(Thy1)_	hAPP_695_/hPSEN1	C57BL/6	Intraneuronal Aβ appears at 6 weeks, while extracellular amyloid plaques are detected in the hippocampus and cortex of 1.5-month-old mice. Cognitive deficits emerge between 3 and 6 months, worsening with age	[491, 492]
3xTg-AD	APP_Swe(Thy1,2)_;tau_P301L(Thy1,2)_;PS1_M146V( PS1)_	hAPP_695_/hTau_4R_/ hPSEN1	C7BL/6;129X1SvJ;129S1/Sv	There is an increase in intracellular Aβ42 levels, synaptic dysfunction and cognitive impairments at 3–4 months. By 6 months of age, extracellular Aβ deposition occurs. By 12 months, tau pathology becomes apparent	[494–496]
TgCRND8xapoE4KI	hAPP_Swe,Ind(PrP)_; hApoE4_( apoE)_	hAPP_695_/h ApoE4	TgCRND8xC57BL/6 J	Comparison to TgCRND8 mice, there is an elevation of IL-1 and GFAP reactivity, accompanied by mild circadian rhythm disturbances	[497]
htau	hTau_(Tau)_	hTau_3R /4R_	C57BL/6 J	At 9 months, tau aggregation and paired helical filaments become evident. These filaments can be isolated as early as 2 months. Hyperphosphorylated tau accumulates from six months onwards, with cognitive deficits emerging by 12 months	[499–502]
Tau Tg	hTau _(PrP)_	hTau_3R0N_	B6SJLF1 x B6D2F1	Mice homozygous for the transgenic insert die at about three months of age. Tau-positive inclusions appear in cortical neurons and brainstem by 6 months of age. By 12 months of age endoneurial space in ventral root axons appears to increase	[503]
rTg4510	hTauP301L_(PrP)_;tTA_(Camk2a)_	hTau_4R0N_	129S6 X FVB	Pretangles appear at 2.5 months, followed by tangle-like inclusions in the cortex at 4 months and the hippocampus at 5.5 months. Spatial memory retention declines between 3 and 5 months	[504, 505]

### AD rat models

Currently, AD rat models are mainly focused on mutant APP.

The rat model expressing mutated hAPP (Swe and Lon mutations; APPK670N/M671L and V717F) (McGill-RThy1-APP) exhibits spatial memory loss at 3 months, working memory impairment from 3 to 6 months, and appearance of Aβ plaques, tau pathology, gliosis, synaptic and neuronal loss at 6 months of age [509, 510].

The rat model expressing hAPP (Swe) / hPSEN1 (ΔE9) (TgF344-AD) shows the development of Aβ plaques, tau pathology, cerebral amyloid angiopathy, gliosis, and neuronal loss at 16 months. Impairments of spatial memory and working memory are observed at 15 and 24 months of age, respectively [511].

The rat model expressing mutated hAPP (Swe and Lon) and PSEN1 (APPK670N, M671L/V717F/PSEN1M146V) (PSAPP) exhibits decreased LTP, abnormal performance in water maze testing, and widespread plaques and gliosis in hippocampus, cortex, olfactory bulb, thalamus, and hypothalamus at 7 months of age. However, reductions in synaptophysin and PSD95 are not observed until 22 months of age [486, 512, 513].

### Non-human primate (NHP) models and AD patient-derived cell and organoid models

The current rodent models have provided valuable tools for understanding AD pathogenesis and exploiting new drugs. However, many mechanisms discovered in rodent models have not been replicated in human cells, and some drugs that were highly effective in animal models have shown poor efficacy in clinical studies. The main reason for this discrepancy may be the great difference between humans and rodents. Currently, efforts to address this issue are mainly focused on the following two directions [471].

#### NHP AD models

NHPs show close genetic homology to humans [484, 485, 487]. They have extended lifespan with similar life stages including childhood, adolescence, adulthood, and aging, and they can perform complex cognitive tests. For these reasons, NHPs may serve as an excellent model of AD. Indeed, multiple AD-like pathological alterations have been detected in aged orangutans, western lowland gorilla and Cynomolgus macaques, which suggest that screening naturally occurring AD in aged monkeys could be a strategy to provide AD NHP models. In addition, transgenic and brain infusion of Aβ and/or tau or their mutant genes may also simulate AD-like pathologies and cognitive deficits. Nonetheless, it is difficult to achieve inbred mating in NHPs, which makes it challenging to ensure experimental reproducibility; and the high cost and the long course of AD also make the experiments using NHPs cost-prohibitive.

#### AD patient-derived cell and organoid models

The human induced pluripotent stem cells (hiPSCs) are emerging as a tool for AD mechanistic studies and preclinical drug testing. The models mainly include inducing differentiation of human embryonic stem cells into neurons or/and into the brain-like tissues in vitro, or using fibroblasts derived from the skin of AD patients and inducing them to acquire stem cell characteristics through reprogramming techniques, followed by further differentiation into human neurons or brain-like tissues to replicate AD models [514–517].

Brain organoids are three-dimensional cellular aggregates derived from iPSCs that recreate different neural cell interactions and tissue characteristics in culture. To produce AD organoid models, the AD-related genes are expressed by viral vectors in human neural progenitor cells (NPC) or NPC differentiated from hiPSC (hiPSC-NPC), and the cells are cultured in specialized 3D culture dishes [518–520]. The NPCs are then induced to differentiate into mature neurons and astrocytes. By co-culturing with microglial cells, microglia migrate and invade into the culture, resulting in 3D brain-like organoid modeling. AD organoid models can recapitulate AD-like Aβ and tau pathologies [521].

Another approach involves reprogramming fibroblasts from FAD patients into FAD-induced pluripotent stem cells (FAD iPSC), followed by differentiation into mature cortical neurons to observe neuronal survival and cellular characteristics [522]. These cells can be further treated with drugs or other methods to study the pathogenesis of AD and screen for potential drugs. This approach is currently being widely used.

## AD diagnosis

The most widely used diagnostic criteria for AD were established in 1984 by the National Institute of Neurological and Communicative Disorders and Stroke and the Alzheimer's Disease and Related Disorders Associations (NINCDS-ADRDA) [5]. In 2011 and 2018, the National Institute of Aging and the Alzheimer's Association (NIA-AA) revised twice the criteria and developed diagnostic guidelines for different stages of AD and mild cognitive impairment (MCI) in clinical settings [523–525]. Specifically, the NIA-AA committee established the inaugural biological definition of AD in 2018, predicated upon the presence of amyloid plaques (A) and tau tangles (T), as opposed to relying solely on symptomatic manifestations. Additionally, the criteria incorporated biomarkers of neurodegeneration (N) to facilitate disease staging. Subsequently, in 2023, a revision of the diagnostic criteria for AD was introduced, taking into consideration recent breakthroughs in understanding the temporal dynamics of biomarkers. This revised framework utilizes these biomarkers to ascertain the progression of the disease. While amyloid and tau continue to hold prominence in the diagnosis and staging of AD, the "N" neurodegeneration marker has been relegated to a secondary position. Within this nascent proposal, currently in its draft phase and intended to elicit feedback from the ADRD research community, the core biomarkers for diagnosis and staging are A and T. Furthermore, this draft scheme acknowledges an expanded array of supplementary markers capable of detecting non-specific disease responses and co-pathologies [526].

### Neuropsychological testing

Cognitive impairment is a significant clinical symptom in neurology, and the assessment of cognitive function primarily relies on neuropsychological testing [527]. Evaluating cognitive function comprehensively allows for a better understanding of patients' cognitive status and characteristics, which plays an essential role in the diagnosis, subtyping, and etiological analysis of cognitive impairment and dementia [528].

The evaluation of cognitive abilities in AD includes memory, language, orientation, visuospatial skills, attention, visual/auditory/tactile perceptions, and executive function. The commonly used neuropsychological assessment tools in the clinic can be categorized as follows: broad assessment scales, such as the Mini-Mental State Examination (MMSE) [529], Montreal Cognitive Assessment (MoCA) [530], Alzheimer's Disease Assessment Scale-Cognitive (ADAS-cog) [531], Hasegawa Dementia Scale (HDS) [532, 533], Mattis Dementia Rating Scale [534, 535], Cognitive Abilities Screening Instrument (CASI) [536], etc.; staging scales, such as the Clinical Dementia Rating (CDR) [537] and Global Deterioration Scale (GDS) [538]; psychiatric behavior rating scales, such as the Hamilton Depression Rating Scale (HAMD) [539] and the Neuropsychiatric Inventory (NPI); discriminatory scales, such as the Hachinski Ischemic Scale. It should be noted that when using the above scales for diagnosing AD, comprehensive judgment should be made by considering clinical manifestations and other auxiliary examination results. Currently, the MMSE, MoCA, and CDR are the most commonly used neuropsychological assessment tools.

### Neuroimaging examinations

Neuroimaging has been used as an auxiliary diagnostic tool for AD since 2007. The main modalities include positron emission tomography (PET) for detecting deposits of Aβ and tau protein, and brain glucose metabolism (FDG PET) [540, 541]; and magnetic resonance imaging (MRI) for detecting brain structural changes [542, 543].

^11^C-Pittsburgh Compound B (^11^C-PIB) was the earliest PET imaging agent used to detect Aβ deposits [544, 545]. It shows Aβ deposition in the frontal, parietal, and temporal lobes of AD patients [546, 547]. These pathological changes can occur before the clinical symptoms of AD, indicating its potential for early diagnosis [548]. Tau PET imaging reveals the presence of pTau tangles in the medial temporal lobe and neocortex, which is highly correlated with the severity of cognitive impairment in AD [549–551]. FDG PET, using ^18^F-FDG as the imaging agent, shows decreased glucose metabolism in bilateral temporal and parietal lobes, as well as the posterior cingulate cortex, in AD patients, and this is correlated with the severity of AD [526, 552]. Structural MRI displays cortical thinning in relevant brain regions of AD patients, along with atrophy in the entorhinal cortex, hippocampus, and posterior cingulate cortex, and a decrease in cortical thickness [553]. Additionally, the application of resting-state functional MRI and diffusion tensor imaging in AD diagnosis is still under research.

### Biomarker analysis

#### The currently used biomarkers for AD diagnosis

In 2018, the NIA-AA classified AD biomarkers into A (amyloid pathology), T (tau pathology), and N (neurodegeneration) [525, 554]. The ATN classification defines AD and describes the changes in each individual at different stages of the disease [555]. The biomarkers reflecting Aβ deposition include CSF Aβ42 levels and PET imaging using Aβ ligands; those reflecting tau pathologies include CSF pTau level and PET imaging using tau ligands; those reflecting neuronal damage/neurodegenerative changes include CSF total and pTau protein levels, CSF neurofilament light chain (NFL) protein level, structural MRI, fluorodeoxyglucose (FDG) PET, and SPECT perfusion imaging, etc. [524, 525]. It is generally believed that the status of Aβ biomarkers determines whether an individual belongs to the AD spectrum, while changes in tau determine whether an individual within the AD spectrum has AD [524, 556]. Using uinoline-derived half-curcumin-dioxaborine (Q-OB) fluorescent probe for detecting Aβ oligomers may reach a preclinical diagnosis of AD [557].

The biomarkers used in AD can be divided into diagnostic and disease progressive biomarkers. The diagnostic biomarkers include Aβ42, total and pTau proteins in CSF, PET imaging using Aβ or tau ligands, and the pathogenic mutations in *APP*, *PSEN1*, and *PSEN2* [525]. These diagnostic biomarkers reflect the underlying pathological processes of AD, but they may not necessarily correlate with disease severity. These biomarkers can be applied for early diagnosis and confirmation of AD. The disease progression biomarkers mainly include brain structural MRI showing hippocampal volume reduction or medial temporal lobe atrophy, FDG PET imaging, etc. Due to their lower specificity, these biomarkers can also exhibit atrophy changes in other conditions such as normal aging and other neurodegenerative diseases. They may not be present in the early stages of AD but can effectively reflect the progression of the disease, thus these markers can be used to monitor the progression of AD [558].

#### Potential periphery biomarkers for AD diagnosis or prediction

The above-mentioned AD diagnostic tools have limitations. Firstly, different doctors may give quite different scores even using the same neuropsychological scale, based on their subject evaluations. Secondly, although the neuroimaging measures have great advantages for the non-invasive and progressive observations, these techniques are currently too expensive to the majority of patients or families. Thirdly, CSF has to be used for measuring the currently recognized molecular biomarkers, however, lumbar puncture is generally not conveniently acceptable by the patients or their family members. Fourthly, *APP*, *PSEN1*, and *PSEN2* mutations only occur in less than 5% of AD patients. Importantly, neurons are terminally differentiated cells with limited regenerating capacity, severer damage may have occurred when the patients come to the hospitals with learning and memory complains.

To deal with these limitations, cross-sectional screening and longitudinal follow-up studies have been carried out to search periphery biomarkers for pre-clinical diagnosis of AD in high-risk populations, such as those at the old age or with T2DM. By dividing the T2DM patients into two groups (i.e., with or without MCI), we found that the T2DM-MCI patients showed GSK-3β activation with olfactory dysfunction and APOE4 genetype compared with T2DM-nMCI populations [21, 25]. Addition of an increased ratio of Aβ1-42/Aβ1-40 to the activated GSK-3β-APOE4-olfactory dysfunction improved the diagnostic efficiency [19]. Further proteomic analysis of the periphery plasma showed that alpha-1-antitrypsin (SERPINA1), major viral protein (PRNP), and valosin-containing protein (VCP) had strong correlation with AD high-risk genes *APP*, *MAPT*, *APOE*, *PSEN1*, and *PSEN2*. Also, the levels of PP2A cancer inhibitor (CIP2A), PRNP, and corticotropin-releasing factor-binding protein (CRHBP) were significantly increased, while the level of VCP was decreased in T2DM-MCI patients compared with the T2DM-nMCI, and these changes were correlated with the MMSE score. Furthermore, the increases of PRNP, CRHBP, VCP, and GSK-3β had the greatest power to identify MCI in T2DM patients [559].

The recent development of blood biomarkers have offered diagnostic and prognostic opportunities that are not feasible using CSF or neuroimaging biomarkers [560]. Plasma Aβ42/Aβ40 ratio and pTau biomarkers targeting the epitopes Thr181 (pTau181), Thr212 (pTau212), Thr217 (pTau217), and Thr231 (pTau231) have each shown high accuracies to identify AD pathology [561–564]. Plasma level of N-terminal containing tau fragments (NTA-tau) increases across the AD continuum, especially during mid-to-late AD stages, and it is closely associated with in vivo tau tangle deposition in AD and its downstream effects [565]. The blood-based brain-derived tau (BD-tau) has been used as a biomarker for identifying Aβ-positive individuals at risk of short-term cognitive decline and atrophy, with implications for clinical trials and implementation of anti-Aβ therapies [566].

As platelets share similarities to neuron biology, it could be an ideal peripheral matrix for biomarkers of neurological disorders. By platelet proteomics, a cluster of altered proteins was identified to be associated with cognitive decline in T2DM patients. Among these proteins, only the increase of optineurin, an autophagy-related protein, was simultaneously correlated with the reduced MMSE score, the activated GSK-3β and the increased Aβ42/40 ratio. Interestingly, the increased optineurin alone could discriminate T2DM-MCI from T2DM-nMCI; and combination of the elevated optineurin with the activated GSK-3β in platelet enhanced the MCI-discriminating efficiency in T2DM patients [22]. Another platelet proteomic analysis in the aged population demonstrated that changes of polyhydroxybutyrate (PHB), ubiquinol-cytochrome C reductase hinge protein (UQCRH), CD63, glycoprotein 1b (platelet), alpha polypeptide (GP1BA), fibronectin (FINC), ras-related protein rap-1A (RAP1A), inositol 1,4,5-trisphosphate receptor type 1/2 (ITPR1/2), and ADAM metallopeptidase domain 10 (ADAM10) could effectively distinguish the cognitively impaired individuals (MCI and AD) from cognitively normal controls; and measuring four reduced proteins (PHB, UQCRH, GP1BA, and FINC) may be enough for predicting cognitive decline in MCI and AD patients [24].

By an integrated analysis of platelet and brain omics from AD patients, we also reported that the changes of 70.3% differentially expressed proteins were consistent in the platelet and the brain. Furthermore, the changes of isocitrate dehydrogenase 3β (IDH3β) and reticulon 1 (RTN1) have a potential diagnostic value for cognitive impairment; Heme oxygenase 2 (HMOX2) and serpin family A member 3 (SERPINA3) may serve as driving molecules in neurodegeneration, and their levels are changed in AD patients [23].

#### Gene analysis

*APP*, *PSEN1*, and *PSEN2* genes have been identified as pathogenic genes in FAD, while *APOE*4 is a high risk gene associated with sporadic AD [7, 11, 93, 567–570]. Therefore, genetic testing can be conducted for cases with a clear family history or early-onset sporadic cases to aid in diagnosis.

Recently, multi-omics data from big cohort along with bioinformatics analysis and machine learning have revealed multiple AD risk genes, providing important information for identifying key pathogenic factors in sporadic AD [11, 571, 572]. New techniques have also been developed to enhance the sensitivity for diagnosing AD [573, 574]. AD risk gene analysis combined with brain functional and pathological imaging, especially PET imaging targeting Aβ and tau, offers possibilities for early diagnosis and intervention in AD [575, 576].

It is worthy of noting that AD is a complex chronic systemic disease, and attention should be given to the diagnosis of different subtypes of AD, which is extremely important for the development of novel disease-modifying drugs.

## AD therapies and development of disease-modifying drugs

Currently, there is still a lack of effective medications or interventions that can cure AD. However, a comprehensive approach involving pharmacological, non-pharmacological, supportive, and caregiving measures can potentially improve the life quality of patients and reduce or delay the progression of AD.

### Drugs being used in the clinic

#### Acetylcholinesterase inhibitors (AChEIs)

AChEIs are primarily used in patients with mild to moderate AD [577]. Acetylcholine (ACh) in the brain plays a role in learning and memory, and dysfunction of the cholinergic system in AD leads to decreased ACh levels. The representative AChEIs include donepezil [578], rivastigmine [579], and galantamine [580], which can increase ACh levels in the brain, enhance synaptic transmission, and improve cognitive functions. They also have some beneficial effects on psychiatric symptoms.

#### NMDAR antagonists

NMDARs play a crucial role in synaptic transmission, a fundamental mechanism for learning and memory. Upregulation of NMDARs in AD results in excitotoxicity characterized by calcium overload and neuronal apoptosis, along with learning and memory deficits. Memantine [581], a NMDAR antagonist, regulates glutamate activity and has certain benefits for memory and psychiatric symptoms [582].

### New disease-modifying drugs under development

The multi-target drugs targeting AD pathologies (i.e., disease-modifying) are under rapid development. Among them, inspiring results have been made on drugs targeting Aβ and tau pathology.

#### Drug development against Aβ

Drug development against Aβ has been the main direction in the field for over two decades. The strategies include active immunity (by injecting Aβ peptide to induce antibody), passive immunity (by directly injecting antibodies against Aβ), use of β- or γ-secretase inhibitors, and blood replacement. Some milestone drugs are stated as follows.

Aducanumab, a monoclonal antibody targeting Aβ developed by Eisai/Biogen, was first conditionally approved by the U.S. Food and Drug Administration (FDA) in June 2021 [583]. This is the first new drug for AD to receive FDA approval since 2003. Following the completion of two randomized, double-blind, placebo-controlled Phase 3 clinical trials by Biogen in 2019, aducanumab was initially declared ineffective, and approximately 40% of participants experienced side effects such as brain swelling and pain [584]. As a result, aducanumab has been the subject of ongoing controversy since its approval. However, the FDA stated that the approval of aducanumab was not based on its efficacy but on its strong and reliable ability to clear Aβ, which may potentially provide clinical benefits. Ongoing large-scale clinical trials are needed to confirm the clinical efficacy of aducanumab.

Lecanemab (also known as BAN2401, marketed under the name Leqembi) is another monoclonal antibody targeting Aβ plaques, developed by Eisai/Biogene. It received expedited approval from the FDA following the release of findings from an 18-month Phase 3 clinical trial [585]. The Phase 3 trial demonstrated a significant 27% reduction in cognitive decline, indicating promising potential for individuals in the early phases of AD [585]. Mechanistically, Lecanemab can block the effects of Aβ/fibrinogen complex on blood clots and the synaptic toxicity in organotypic culture [586].

Donanemab (also known as N3pG) is a humanized IgG1 monoclonal antibody developed by Lilly from the mouse mE8-IgG2a. Unlike other therapeutic Aβ antibodies, donanemab specifically targets Aβ(p3-42), which is a pyroglutamate form of Aβ found in aggregated amyloid plaques. Its high affinity for deposited amyloid plaques differentiates it from antibodies that have low affinity for these plaques. The rationale behind donanemab is to directly target and clear existing amyloid burdens in the brain rather than solely preventing new plaque deposition or growth of existing plaques [587]. Donanemab also slows the rate of accumulation of tau NFTs in the frontal cortex and other brain regions [588]. In June 2021, Donanemab received the Breakthrough Therapy designation from the FDA to expedite its development and review process. However, in January 2023, the FDA rejected the accelerated approval application for donanemab, citing the need for additional safety data. On May 4, 2023, Lilly announced positive top-line results from the Phase 3 study Trailblazer-ALZ2 [589]. Treatment with Donanemab significantly slowed the decline on the primary outcome measure of integrated Alzheimer's Disease Rating Scale (iADRS) by 40% and demonstrated improvement in all secondary clinical endpoints [590].

#### Drug development against tau

The level of abnormal tau in AD brain is positively correlated with the degree of clinical dementia. The Braak-Braak classification for AD progression is based on the spreading of pTau/tangles in the specific brain areas. Animal studies have shown that tau depletion mitigates the toxic effects of Aβ. In addition, pTau is the main component of degenerative neurons in the autopsy AD brains. Therefore, targeting tau may be promising in AD drug development.

Currently, drug development strategies against tau include immunotherapies (antibodies/vaccines), suppressing the synthesis (siRNA, miRNA, ASO), inhibiting the aggregation (such as LMTX by TauRx Therapeutics), and regulating post-translational modifications.

Semorinemab (RG 6100) is a humanized IgG4 monoclonal antibody targeting the N-terminal of tau (amino acid residues 6–23). A phase 2 clinical trial showed that after 49 weeks of treatment, patients in the semorinemab group experienced 43.6% less cognitive decline than those in the placebo group. The *K*_d_ value of semorinemab binding to human tau protein is 3.8 nM [591, 592]. As the NFTs formed by pTau are located inside the neural cells, the antibodies/vaccines targeting tau proteins need to penetrate both BBB and the neural cell membrane to exert functions. The latter is an additional barrier compared with Aβ-vaccine therapies.

BIIB080 (IONIS-MAPT), an antisense oligonucleotide (ASO) co-developed by Ionis and Biogen, has shown positive results in a clinical 1b trial. The data showed that BIIB080 reduced soluble tau protein in the CSF of early AD patients in a dose-dependent and sustained manner. BIIB080 also reduced pathological tau accumulation evaluated by PET imaging. These results were presented at the International Conference on Alzheimer's and Parkinson's Diseases (AD/PD™2023) [593]. Inhibiting tau synthesis (such as ASO) should be promising in decreasing total tau level though it is generally recognized that tau accumulation in the AD brain is not due to an increased transcription or translation.

LMTX, a tau aggregation inhibitor, has passed Phase 3 [594]. By comparing the cognitive abilities and measuring the rate of brain atrophy, researchers found that the LMTX monotherapy led to significant improvements in cognitive performance and a significant reduction in the rate of brain atrophy at doses of 100 mg or 4 mg twice daily [595].

As abnormal post-translational modifications are the major cause of tau aggregation in AD, modulating the enzymes involved in different post-translational modifications has attracted great attention. However, as the enzymes involved in tau post-translational modifications have broad substrates, either upregulating or downregulating the enzymes will unavoidably cause side effects, especially in chronic diseases like AD.

Induced-proximity technologies are newly developed approaches that do not change the enzyme activity. These approaches include proteolytic targeting chimera [596, 597] and deubiquitinating enzyme targeting chimeras [598], both promoting degradation of total tau. To specifically target the pTau which is the major component of the NFTs, we recently developed dephosphorylation targeting chimera, which can efficiently and specifically promote tau dephosphorylation with restoration of the biological function of tau [29]. A similar approach has been reported recently by Crews’ group [30].

#### Others

Among many other disease-modifying drug attempts, the National Medical Products Administration (NMPA) in China has conditionally approved the market application of a new drug called sodium oligomannate (also known as GV-971) for the treatment of mild to moderate AD patients [599]. GV-971 is a low-molecular-weight acidic oligosaccharide compound derived from marine brown algae. Although there are still many uncertainties, this innovative drug has provided a new therapeutic option for AD [600].

### Management of psychiatric symptoms

Many AD patients experience psychiatric symptoms during certain stages of the disease, such as hallucinations, delusions, depression, anxiety, agitation, and sleep disturbance. Antidepressant [601] and antipsychotic medications can be used to manage these symptoms [602]. Selective serotonin reuptake inhibitors such as fluoxetine, paroxetine, sertraline, and citalopram are commonly used as antidepressants [601]. Atypical antipsychotics such as risperidone, olanzapine, and quetiapine are often prescribed as antipsychotic agents [602]. The principles for the use of these medications are as follows: starting with a low dose and increasing the dose gradually, with longer intervals between dose increments; using the minimum effective dose whenever possible; individualizing treatment, and keeping in mind drug interactions.

### Non-pharmacological treatments

Non-pharmacological treatments encompass dietary adjustments [603] (e.g., Mediterranean diet or time-restricted feeding) [604, 605], cognitive training [606], physical exercise [607, 608], music therapy [609], etc. These measures are more readily accepted by patients and their families, and may have a preventive effect or delay the decline in daily functioning of patients, improving their life quality.

In addition, research on specific modulation of brain regions and neural circuits (such as deep brain stimulation, transcranial magnetic stimulation and phototherapy), stem cells, gene editing, etc., has attracted great attention.

### Supportive care

Severe AD patients experience a significant decline in daily activities, often leading to complications such as malnutrition, pulmonary or urinary tract infections, ulcers, etc. [610]. The course of AD is typically around 5 to 10 years, and some patients survive even longer. Therefore, it is important to strengthen supportive care and provide symptomatic treatment. In addition, a healthy lifestyle is beneficial for the prevention of AD.

## Conclusion

In this review, we updated the advances in AD research from basic mechanism studies to clinic diagnosis and drug development. Continued collaborative efforts and interdisciplinary research will be important in unraveling the complexities of AD and ultimately making a significant impact on the patients affected by this devastating disease.

## Data Availability

Not applicable.

## Abbreviations

AD: Alzheimer’s disease
Aβ: β-Amyloid
NFT: Neurofibrillary tangle
PHF: Paired helical filament
APOE: Apolipoprotein E
APP: Amyloid precursor protein
CSF: Cerebrospinal fluid
NMDA: N-methyl-D-aspartate
FTDP-17: Parkinsonism linked to chromosome 17
GSK-3β: Glycogen synthase kinase-3β
MAP: Microtubule-associated protein
LTP: Long term potentiation
PSP: Progressive supranuclear palsy
CREB: CAMP-response element binding protein
mTORC1: Mammalian target of rapamycin kinase complex 1
ESCRT: Endosomal Sorting Complex Required for Transport
PDPK: Proline-directed protein kinases
ERK: Extracellular signal-related protein kinase
Cdk: Cyclin-dependent kinase
PKA: Cyclic-AMP-dependent protein kinase
PKC: Protein kinase C
PRR: Proline-rich region
SUMO: Small ubiquitin-like modifier
MBD: Microtubule-binding domain)
AGE: Advanced glycation end product
AEP: Asparagine endopeptidase
LRP: Low-density lipoprotein receptor-related protein
BACE1: β-Site APP cleaving enzyme-1
IDE: Intracellular insulin-degrading enzyme
MMPs: The matrix metalloproteases
VLDL-R: Very low-density lipoprotein receptor
LDL-R: Low-density lipoprotein receptor
NF-κB: Nuclear factor kappa-B
ADAM: α-Disintegrin and metalloproteinase
RAGE: Receptors for advanced glycation end products
BBB: Blood-brain barrier
ADDLs: Aβ-derived diffusible ligands
HSV: Herpes simplex virus type
HHV: Human herpesvirus
HCV: Hepatitis C virus
LPS: Lipopolysaccharides
TLRs: Toll-like receptors
TREM: Myeloid cell trigger receptor
NHPs: Non-human primates
hiPSCs: Human induced pluripotent stem cells
NPC: Neural progenitor cells
T2DM: Type 2 diabetes mellitus
CRHBP: Corticotropin-releasing factor-binding protein
